# The Hill function is the universal Hopfield barrier for sharpness of input–output responses

**DOI:** 10.1073/pnas.2318329121

**Published:** 2024-05-24

**Authors:** Rosa Martinez-Corral, Kee-Myoung Nam, Angela H. DePace, Jeremy Gunawardena

**Affiliations:** ^a^Department of Systems Biology, Harvard Medical School, Boston, MA 02115; ^b^HHMI, Boston, MA 02115

**Keywords:** coarse-graining, Hill function, Hopfield barrier, linear framework, model assumptions

## Abstract

The Hill functions, $H_{h}\left(x\right)=x^{h}/\left(1+x^{h}\right)$, have been widely used in biology for over a century but, with the exception of $H_{1}$, they have had no justification other than as a convenient fit to empirical data. Here, we show that they are the universal limit for the sharpness of any input–output response arising from a Markov process model at thermodynamic equilibrium. Models may represent arbitrary molecular complexity, with multiple ligands, internal states, conformations, coregulators, etc, under core assumptions that are detailed in the paper. The model output may be any linear combination of steady-state probabilities, with components other than the chosen input ligand held constant. This formulation generalizes most of the responses in the literature. We use a coarse-graining method in the graph-theoretic linear framework to show that two sharpness measures for input–output responses fall within an effectively bounded region of the positive quadrant, $\Omega_{m}\subset {\left(R^{+}\right)}^{2}$, for any equilibrium model with m input binding sites. $\Omega_{m}$ exhibits a cusp which approaches, but never exceeds, the sharpness of $H_{m}$, but the region and the cusp can be exceeded when models are taken away from thermodynamic equilibrium. Such fundamental thermodynamic limits are called Hopfield barriers, and our results provide a biophysical justification for the Hill functions as the universal Hopfield barriers for sharpness. Our results also introduce an object, $\Omega_{m}$, whose structure may be of mathematical interest, and suggest the importance of characterizing Hopfield barriers for other forms of cellular information processing.

## Significance

Biological systems can process information without expending energy, and the limit to what can be achieved in this way is known as a Hopfield barrier. We characterize this barrier for the sharpness of input–output responses, making typical assumptions about the underlying molecular mechanisms. If an input ligand binds at N sites, we show that the Hopfield barrier for sharpness is the Hill function with coefficient N, irrespective of the molecular details. This provides a biophysical justification for the widely used Hill function, which was introduced over a century ago only as an empirical fit to data. Furthermore, when data exceed the sharpness barrier, the strong conclusion may be drawn that the underlying mechanism is expending energy.

The Hopfield barrier for an information processing task is the fundamental upper bound to how well that task can be implemented by a mechanism that operates at thermodynamic equilibrium (1). The only way to exceed this barrier is by expending energy to maintain a steady state away from thermodynamic equilibrium. The existence of such barriers was first pointed out by John Hopfield in his pioneering work on kinetic proofreading for reducing errors in biosynthetic processes like DNA replication (2). The broader principle just outlined, applicable to any information processing task, is named in his honor (1).

In the present paper, we determine the universal Hopfield barrier for the sharpness of steady-state input–output responses. Such responses have been widely used in biochemistry, molecular biology, physiology, and pharmacology to quantitatively describe the functional behavior of biological systems, such as receptors, ion channels, enzymes, transporters, allosteric systems, signaling pathways, gene-regulatory systems, tissues, etc, which interact with an input ligand to produce some output behavior; see *SI Appendix*, Table S1. Sharpness, or ultrasensitivity, refers to the amount of output change for a given change in the input, and is often measured by reference to the family of Hill functions,

[1] $$\begin{array}{c} H_{h}\left(x\right)=\frac{x^{h}}{1+x^{h}}\;. \end{array}$$

Here, h>0 is the Hill coefficient and x is the normalized concentration of the input ligand. The Hill coefficient is frequently quoted as a measure of sharpness, with positive sharpness corresponding to h>1 and negative sharpness to h<1. When h=1, $H_{1}\left(x\right)$ is the classical Michaelis–Menten input–output response (3), which is the baseline for the absence of sharpness. Hill functions are also frequently used to represent input–output responses in dynamical system models, where their sharpness underlies the emergence of multiple steady states or limit cycle oscillations (4, 5).

Because Hill functions are so widely used, it is sometimes forgotten that they have no mechanistic justification when h≠1 (6, 7). Unlike the Michaelis–Menten response $H_{1}\left(x\right)$, which was originally derived to explain enzyme kinetics and has been found in many other contexts (8), the Hill functions are merely a convenient family of rational functions which Archibald Vivian Hill selected to fit data on the oxygen-binding response of hemoglobin (9). One of the main results of this paper is to give a rigorous biophysical justification for the Hill functions.

**Fig. 1.**
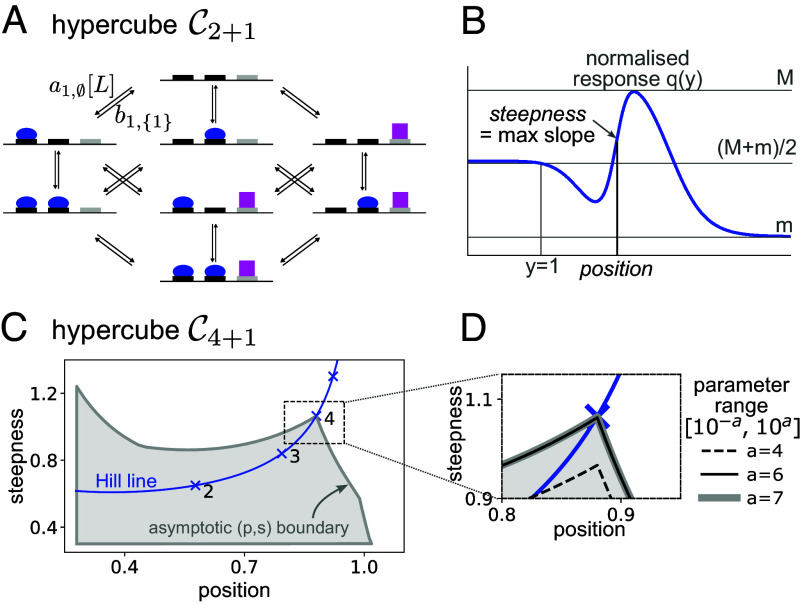
Hypercube-structure graph, definition of position and steepness, and position-steepness region. (*A*) Linear framework graph with the hypercube structure, $C_{2+1}$, representing the binding to a biomolecule of one ligand (L, blue oval) to two sites and a second ligand (magenta square) to a third site. Only two edge labels are shown for clarity, with the binding edge label containing a term, [L], for the free concentration of ligand L. This graph could describe a gene regulation system in which L is a transcription factor that recruits RNA Polymerase to a promoter (1, 10). (*B*) Normalized input–output response q(y) (blue curve), where the normalization procedure for y, depicted in gray font, ensures that q(1)=(M+m)/2 (Eq. **10**). Steepness is defined as the maximal unsigned slope of q(y), and position as the smallest value of y for which the steepness is attained (Eq. **11**). (*C*) Asymptotic (p,s) region for the hypercube $C_{4+1}$, obtained by random sampling of parameters over increasing ranges until the boundary of the occupied region stabilizes (see *Inset* in panel *D*). The input ligand binds at four sites, the output is the steady-state probability of the fifth site being occupied by its ligand, and the graph is at thermodynamic equilibrium. The region has been truncated to the left and below to focus on the area of interest around the *Hill line* (blue), which is the locus of (p,s) points for the Hill functions (Eq. **1**), with the integer Hill coefficients marked. (*D*) Expanded view of the region in panel *C* in the vicinity of the cusp, showing the asymptotic stabilization as the parametric range increases (see also *SI Appendix*, Figs. S4 and S5).

We provide an overview of our approach and results here before explaining the technical details below. We specify input–output responses using the linear framework, an approach to Markov processes based on directed graphs with labeled edges (11, 12); for up-to-date reviews, see refs. 13 and 14. In this approach, graph vertices represent molecular states, directed edges represent transitions and edge labels represent transition rates. Fig. 1*A* shows a graph with a hypercube structure, denoted $C_{2+1}$, which represents the binding and unbinding of two ligands to three sites on a biomolecule. We use structure to refer to vertices and edges only, disregarding labels. Hypercube-structure graphs frequently underlie models of input–output responses (15–18), including several in *SI Appendix*, Table S1. Edge labels can include terms, such as concentrations of binding ligands, that describe the interaction between the graph and its environment (Fig. 1*A*). The core assumptions about ligands are detailed below.

The linear framework allows the steady-state probabilities of graph vertices to be calculated as rational algebraic functions of the labels (Eq. **4**). Importantly, this can be done whether or not the steady state is one of thermodynamic equilibrium, a property that is determined by the edge labels. An output response can then be defined as a nonnegative linear combination of the steady-state probabilities and considered as a function of the concentration of a chosen input ligand, with all other concentrations kept constant (Eq. **5**).

In previous work, we introduced two intrinsic, nondimensional measures, position and steepness to quantify the sharpness of such an input–output response (1), as described in Fig. 1*B*. By sampling parameter values appropriately, we plotted the two-dimensional position-steepness, or (p,s), regions for various input–output responses on hypercube-structure graphs with different numbers of input binding sites, assuming the corresponding systems were at thermodynamic equilibrium (1, 10). Fig. 1*C* shows part of the (p,s) region for a hypercube-structure graph similar to that in Fig. 1*A* but with four binding sites for the input ligand.

We found that these (p,s) regions exhibit four characteristic properties. First, the regions have an asymptotic boundary: if the range over which the model parameters are sampled is steadily increased, the boundary of the (p,s) region stabilizes (Fig. 1*D*), giving rise to the asymptotic boundary and the asymptotic region (Fig. 1*C*). Second, the boundary encloses a region that is “effectively bounded” in the positive quadrant. The (p,s) region is not bounded in the whole positive quadrant $R^{+}\times R^{+}$; there are wings that become asymptotic to the axes. These wings are not shown in Fig. 1*C*; they are not the focus of this paper and appear not to be biologically relevant. However, given a>0, no matter how small, that part of the (p,s) region which falls within [a,∞)×[a,∞) is bounded, which is what we mean by “effectively bounded.” Third, these (p,s) regions exhibit a cusp that falls on the Hill line, the locus of (p,s) points for the Hill functions. The tip of the cusp lies below the (p,s) point with Hill coefficient equal to the number of input binding sites and approaches this Hill point more closely as the parametric range increases (Fig. 1*D*). In other words, if the system has m binding sites for the input ligand, the (p,s) point for $H_{m}$ acts as a barrier to sharpness. Note the importance of using two sharpness measures to draw this conclusion: each one of the measures can individually exceed the corresponding value for $H_{m}$ but they cannot both do so simultaneously (Fig. 1*C*). Fourth, parameter values can be found away from thermodynamic equilibrium whose (p,s) points lie above and to the right of the (p,s) point of $H_{m}$. This confirms that $H_{m}$ is the Hopfield barrier for sharpness of input–output responses on hypercube-structure graphs like those in Fig. 1*A*.

Results of this kind already inform the interpretation of experimental data. If the data fall outside the (p,s) region, then no model of this kind can account for the data, no matter what parameter values are chosen, and this can be asserted without fitting the model to the data (10). Of course, it is necessary to know the number of binding sites to draw this conclusion. If that number is not known with certainty, the result still provides a lower bound for the number of binding sites that are needed to account for the data with a model at thermodynamic equilibrium. However, the scope of such conclusions is limited by the underlying hypercube structure. Much greater molecular complexity may actually be present, such as coregulators, conformations, internal states, modifications, etc. (*SI Appendix*, Fig. S1). A different model will typically yield a different (p,s) region (*SI Appendix*, Fig. S6) and this region may be able to account for the data.

We will show here that this limitation may be overcome for sharpness at thermodynamic equilibrium. We use a method of coarse-graining in the linear framework to show that there is a universal bounded region that contains the (p,s) point for the input–output response of any Markov process model at thermodynamic equilibrium and this region exhibits the same four properties described above. The model may be arbitrarily complicated, subject to the core assumptions detailed below. In particular, the Hill function emerges as the universal, model-independent Hopfield barrier for sharpness.

Most studies in the biological literature focus on specific models, and it is rare to be able to make a rigorous claim about all models within a large and widely used class, such as the Markov process models studied here. The universality that we have uncovered for sharpness may potentially hold more widely and suggests new directions to explore in the mathematics and biophysics of cellular information processing (*Discussion*).

## Results

### Graphs, Markov Processes, and Input–Output Responses.

The linear framework was introduced in refs. 11 and 12 and reviewed in refs. 13, 14, and 19; the *Materials and Methods* and *SI Appendix* provide more details. Under the reservoir assumptions defined below, linear framework graphs are equivalent to finite-state, continuous-time, time-homogeneous Markov processes that have infinitesimal generators (12, Theorem 4). The graph specifies the master equation of the Markov process (*Materials and Methods*, Eq. **13**), which is a linear differential equation from which the framework acquires its name. Graph vertices represent the states of the Markov process. There is an edge between two vertices when the infinitesimal rate for this transition is positive, in which case this positive rate becomes the edge label, with dimensions of (time)^−1^. Vertices are typically denoted by 1,⋯,n, edges by i→j, and labels by ℓ(i→j). Edge labels can contain terms that describe the interaction between the graph and its environment (Fig. 1*A*). *SI Appendix*, Fig. S1 shows some of the molecular complexity that may be accommodated within the graph formalism. From now on, we will refer interchangeably to graphs and their corresponding Markov processes.

The use of graphs to study Markov processes has its roots in the pioneering work of Hill (20) and Schnakenberg (21). It is rarely seen in the Markov process literature and has only occasionally appeared in the biophysics literature (22), until the development of the linear framework (23–26). The main distinction in the linear framework approach is to treat the graph as a mathematical object in its own right, in terms of which results can be formulated, which, as we will see here, can accommodate some of the molecular complexity found in biology.

We use “ligand” to refer to any component in the environment that interacts with the graph through binding and unbinding, like those represented by the blue oval and magenta square in Fig. 1*A*. Depending on the context, such as gene regulation, a ligand may be a transcription factor, an enzyme complex like RNA Polymerase, a coregulator like Mediator, a nucleosome, etc (27). Ligand binding is assumed to follow mass action and to be first order, so that a binding edge label acquires a term for the free ligand concentration (Fig. 1*A*). Ligands are assumed not to engage in activities outside the graph, such as oligomerization; such activities may be accommodated (28) but complicate the arguments given here. Most importantly, ligands are assumed to be present in sufficient quantity that binding does not appreciably change their free concentration. This reservoir assumption, which is implicitly made in all treatments of input–output responses, is similar to the assumption in classical thermodynamics of a heat bath, with which energy can be exchanged without altering the temperature. First-order binding and reservoirs are the core assumptions that underlie all the models and results of this paper; they are commonly used in the literature, not always explicitly.

The linear framework enables the steady-state (s.s.) probability, $u_{i}^{*}\left(G\right)$, of vertex i of graph G to be calculated as a rational function of the edge labels (11, 12). Recall that G is strongly connected if any two distinct vertices, i≠j, are connected by a directed path, $i=i_{1}\to i_{2}\to \cdots \to i_{k}=j$. Provided G is strongly connected, there is a unique s.s., which is described up to a proportionality constant by the vector, ρ(G), with components,

[2] $$\begin{array}{c} \rho_{i}\left(G\right)=\sum_{T\in \Phi_{i}\left(G\right)}\prod_{u\to v\in T}\ell \left(u\to v\right)\;. \end{array}$$

Here, $\Phi_{i}\left(G\right)$ is the set of spanning trees of G that are rooted at i. A spanning tree is a subgraph of G that includes every vertex (spanning), has no cycles when edge directions are ignored (tree), and has only one vertex with no outgoing edge (the root). The s.s. probability is recovered from Eq. **2** by normalizing, as in Eq. **4**.

Eq. **2** shows that s.s. probabilities depend on all the edge labels in the graph and are subject to a combinatorial explosion even for relatively small graphs, which arises from having to enumerate all spanning trees: the structure $C_{4}$, for example, has 42,467,328 spanning trees rooted at each vertex (29). However, a substantial simplification occurs if G can reach a s.s. of thermodynamic equilibrium (t.e.). A graph G is at t.e. if two conditions are satisfied. First, G is reversible, so that if i→j, then the reverse transition, j→i, is also present. Second, detailed balance holds, so that any pair of reversible edges, i⇋j, is independently in flux balance: $u_{i}^{*}\left(G\right)\ell \left(i\to j\right)=u_{j}^{*}\left(G\right)\ell \left(j\to i\right)$. Reaching a s.s. in which detailed balance holds is equivalent to the following cycle condition on the labels of a reversible graph. Let P be any path of reversible edges, $P:\;i_{1}⇋i_{2}⇋\cdots ⇋i_{k}$, and let μ(P) denote the product of the label ratios along P,

[3] $$\begin{array}{c} \mu \left(P\right)=\left(\frac{\ell (i_{1}\to i_{2})}{\ell (i_{2}\to i_{1})}\right)\cdots \left(\frac{\ell (i_{k-1}\to i_{k})}{\ell (i_{k}\to i_{k-1})}\right)\;. \end{array}$$

The cycle condition requires that μ(P)=1 whenever the path is a cycle, with $i_{k}=i_{1}$. The quantity logμ(P) is interpreted in stochastic thermodynamics as the entropy generated along P (30), so that t.e. corresponds to there being no entropy generation over cycles in G.

At t.e., an alternative vector, μ(G), may be used to calculate s.s. probabilities. Choose a reference vertex, which we will index as 1. In principle, this can be any vertex but it will be convenient to choose one in which no input binding site is bound (*Materials and Methods*). Now choose any path, $P_{i}$, of reversible edges from 1 to i; and let $\mu_{i}\left(G\right)=\mu \left(P_{i}\right)$. The cycle condition ensures that this is well defined. The s.s. probability can then be determined by normalizing,

[4] $$\begin{array}{c} u_{i}^{*}\left(G\right)=\frac{\mu_{i}\left(G\right)}{\mu_{1}\left(G\right)+\cdots +\mu_{n}\left(G\right)}\;. \end{array}$$

This normalization can be done either with μ(G) at t.e., as shown in Eq. **4**, or with ρ(G) in the general case. Either way, the resulting expression is a rational function of the edge labels.

When G can reach t.e., the quantities $\log\mu_{i}\left(G\right)$ can be interpreted in terms of the free energy of vertex i relative to the reference vertex 1. Eq. **4** then recovers the classical formula of equilibrium statistical mechanics: the denominator is the partition function for the grand canonical ensemble and the terms $\mu_{i}\left(G\right)$ provide the Boltzmann factors. A key advantage of the linear framework is that it reduces to equilibrium statistical mechanics at t.e. but it also enables s.s. probabilities to be exactly calculated away from t.e. by using Eq. **2**.

Input–output responses on G may now be defined by choosing some ligand as input. We denote its concentration by x. We assume that x is changed quasi-statically—in small increments and sufficiently slowly that the graph relaxes back to a s.s. after each change—which fits the conditions under which input–output responses have been measured (*SI Appendix*, Table S1). The concentrations of any other ligands are assumed to be held constant. The output can be any nonnegative linear combination of s.s. probabilities, considered as a function of x,

[5] $$\begin{array}{c} r\left(x\right)=\sum_{1\leq i\leq n}\lambda_{i}u_{i}^{*}\left(G\right)\;,\;\;\;\;0\leq \lambda_{i}\leq 1\;. \end{array}$$

The restrictions ensure that r(x) is nondimensional and normalized to lie in [0,1]. It follows from Eqs. **2** and **4** that r(x) is a rational function of x.

### Coarse-Graining.

In this section, we will show that if G is any strongly connected, reversible graph that reaches t.e., and r(x) is any input–output response on G, then r(x) can be rewritten as an input–output response on some reversible substructure of the hypercube $C_{m}$, where m is the number of input binding sites, with edge labels that satisfy the cycle condition. This result begins to explain how universality arises at t.e.: no matter how complex the input–output response, it is mathematically equivalent to one that involves only the binding and unbinding of the input ligand. This rewriting requires finding edge labels for the substructure of $C_{m}$, as well as the appropriate coefficients for its input–output response. The coarse-graining strategy introduced in ref. 31 provides the necessary approach. We apply it here with further details in the *Materials and Methods*. To avoid trivial special cases, we assume from now on that m>1.

**Fig. 2.**
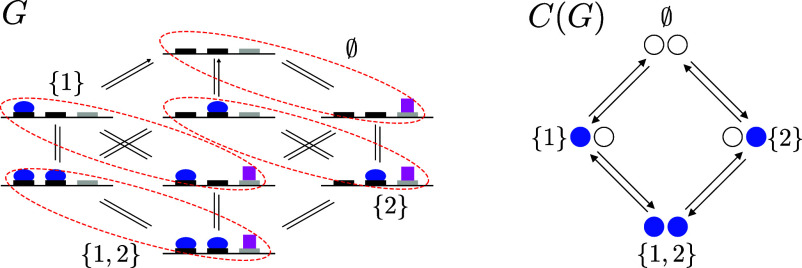
Coarse-graining, showing only graph structures. On the *Left*, the structure G, from Fig. 1*A*, is being coarse-grained, as described in the text, with the blue oval as the input ligand L. The vertices of G are partitioned into subsets (red, dashed ovals) corresponding to the patterns of binding of L to m=2 sites; the magenta square is ignored. The input binding sites are indexed 1 and 2 from left to right and the subsets of sites are indexed in set notation ∅,{1},{2},{1,2}. The resulting coarse-grained structure, C(G), is shown on the *Right*, with vertices indexed by the corresponding subsets. In this case, the full structure of the hypercube $C_{2}$ is recovered. The edges and labels of C(G) are explained in the text and *SI Appendix*.

Let L be the input ligand and let ν(G) denote the set of vertices of G. Coarse-graining does not require G to satisfy the cycle condition, although we will make this assumption later. Coarse-graining starts from any partition of the vertices of G into disjoint subsets and constructs a linear framework graph, C(G), whose vertices are the subsets of the partition. We choose the partition given by collecting together those vertices with the same pattern of binding of L. Binding patterns are indexed by subsets S⊆{1,⋯,m}. Let $G_{S}\subseteq \nu \left(G\right)$ contain those vertices i∈ν(G) such that, if s∈S, then L is bound at s in vertex i, but if s∉S, then L is not bound at s in vertex i. The vertex i may have many other features as a vertex of G in addition to the sites bound by L, but this coarse-graining ignores them. There is an edge in C(G), $w\to_{C(G)}z$ if, and only if, there is an edge in G, $i\to_{G}j$, for some vertex $i\in G_{w}$ and some vertex $j\in G_{z}$. It follows that the vertices and edges of C(G) are those of the hypercube structure $C_{m}$. But C(G) may not be all of $C_{m}$. This can happen because of mutual exclusion (*SI Appendix*, Fig. S1*B*), in which some vertices of $C_{m}$ are not reached, or because of ordering (*SI Appendix*, Fig. S1*C*), in which some edges of $C_{m}$ are not used. Accordingly, the structure of C(G) is generally only a substructure of $C_{m}$. Fig. 2 shows an example of coarse-graining in which C(G) is all of $C_{2}$.

It can be shown that labels may be assigned to the edges of C(G) in essentially only one way (*Materials and Methods*, Eq. **14**), such that C(G) satisfies the cycle condition and the following coarse-graining equation holds (31),

[6] $$\begin{array}{c} u_{w}^{*}\left(C\left(G\right)\right)=\sum_{i\in G_{w}}u_{i}^{*}\left(G\right)\;. \end{array}$$

Eq. **6** is what would be expected from a coarse-graining at s.s. Eq. **5** may now be rewritten as,

[7] $$\begin{array}{c} r\left(x\right)=\sum_{S\in \nu (C(G))}\left(\sum_{i\in G_{S}}\lambda_{i}\chi_{i}\right)u_{S}^{*}\left(C\left(G\right)\right)\;, \end{array}$$

where $\chi_{i}$ can be seen from Eq. **6** as the s.s. probability of i conditioned on the subset $G_{S}\subseteq \nu \left(G\right)$,

[8] $$\begin{array}{c} \chi_{i}=\frac{u_{i}^{*}\left(G\right)}{\sum_{j\in G_{S}}u_{j}^{*}\left(G\right)}\;. \end{array}$$

The terms $\chi_{i}$ may be extremely complicated in general, as they summarize the other features that are present in G. The key point, however, is that if G does satisfy the cycle condition, then the $\chi_{i}$ do not depend on x (*Materials and Methods*). It follows that, provided that G satisfies the cycle condition, Eq. **7** expresses r(x) as a valid input–output response on a graph that is a substructure of $C_{m}$, as claimed above. Let us call this graph $G_{m}$.

A universal (p,s) region can now be generated by sampling not only the labels of $G_{m}$ but also the coefficients $\lambda_{i}$ (Eq. **5**) that appear in the input–output responses on $G_{m}$. However, a further simplification arises because r(x) has the following rational structure (*Materials and Methods*),

[9] $$\begin{array}{c} r\left(x\right)=\frac{\alpha_{0}+\alpha_{1}x+\cdots +\alpha_{l}x^{l}}{\beta_{0}+\beta_{1}x+\cdots +\beta_{l}x^{l}}\;, \end{array}$$

where l is the maximum number of sites bound by L, so that 1≤l≤m; the denominator coefficients are all positive, $0<\beta_{i}$; and the numerator coefficients are nonnegative and not greater than the corresponding coefficients in the denominator, $0\leq \alpha_{i}\leq \beta_{i}$. It can be shown that any choice of $\alpha_{i}$ and $\beta_{i}$ that satisfies these conditions corresponds to an equilibrium input–output response (*SI Appendix*), so that Eq. **9** exactly describes the equilibrium input–output responses of Markov process systems with m input binding sites.

Eq. **9** shows that the rational structure of an equilibrium input–output response is largely independent of the graph G from which it is derived. G determines the coefficients, $\alpha_{i},\beta_{i}$, but the degree in x of the denominator of r(x), namely l, depends only on the maximum number of sites bound by the input, irrespective of the complexity of G. The rational structure of Eq. **9** is a preliminary mathematical expression of the universal Hopfield barrier and is the basis for analyzing sharpness below.

The nondependence of $\chi_{i}$ on x, which is crucial for the structure of r(x) described in Eq. **9**, breaks down emphatically if G does not satisfy the cycle condition. The resulting r(x) can then no longer be an input–output response on some substructure of $C_{m}$. The algebraic structure of nonequilibrium input–output responses is strikingly different, as we will see below.

### Intrinsic Measures of Sharpness.

To define measures of sharpness, it is necessary to normalize the input–output response. The output value is normalized already in the light of Eq. **5**. Since there is no naturally independent quantity against which to normalize the input concentration, x, its normalization has to be intrinsically determined for each response. The input–output responses allowed by Eq. **9** can be nonmonotonic and complicated (Fig. 1*B*). Accordingly, we choose the normalization value, denoted $x_{0.5}$, to be the smallest positive value of x at which the response is halfway between its supremum and its infimum, which exist because 0≤r(x)≤1. More precisely, we define (Fig. 1*B* and *SI Appendix*, Fig. S2),

[10] $$\begin{array}{c} \begin{array}{cc} m(r) & =\underset{x\in [0,\infty )}{\inf}r\left(x\right)\;,\;\;\;M\left(r\right)=\underset{x\in [0,\infty )}{\sup}r\left(x\right)\\ x_{0.5} & =\underset{x\in (0,\infty )}{\inf}\left\{x\;|\;r\left(x\right)=\frac{m(r)+M(r)}{2}\right\}\;. \end{array} \end{array}$$

We explicitly choose $x_{0.5}>0$. This can always be done because, even if r(0)=(m(r)+M(r))/2, there must be x>0 for which r(x) has the same value. The normalized response, q(y), where $y=x/x_{0.5}$, is then defined by $q\left(y\right)=r\left(yx_{0.5}\right)$. Note that $x_{0.5}$ depends on r.

Following normalization, the two intrinsic measures of sharpness are the supremum of the absolute value of the derivative of q(y), which we call steepness and denote s(r), and the smallest y value that attains the supremum, which we call position and denote p(r). The supremum is attained at a finite value of y (*SI Appendix*), so that,

[11] $$\begin{array}{c} s\left(r\right)=\max_{y\in [0,\infty )}|\frac{\mathrm{dq}}{\mathrm{dy}}|\;\;,\;\;\;p\left(r\right)=\min_{y\in [0,\infty )}\;\text{argmax}|\frac{\mathrm{dq}}{\mathrm{dy}}|\;. \end{array}$$

Because of the dependence of $x_{0.5}$ on r, position and steepness are scale invariant: p(r(cx))=p(r) and s(r(cx))=s(r), for any scale factor c>0. If we denote by $s^{u}\left(r\right)$ and $p^{u}\left(r\right)$ the unnormalized versions of steepness and position, obtained using dr/dx in place of dq/dy in Eq. **11**, then the relationship between the normalized and unnormalized versions is given by a complementary scaling by $x_{0.5}$: the steepness is multiplied and the position is divided,

[12] $$\begin{array}{c} s\left(r\right)=s^{u}\left(r\right)x_{0.5}\;\;,\;\;\;p\left(r\right)=\frac{p^{u}\left(r\right)}{x_{0.5}}\;. \end{array}$$

This relationship will be helpful to interpret (p,s) regions below.

### Universal Position-Steepness Region and the Hopfield Barrier.

**Fig. 3.**
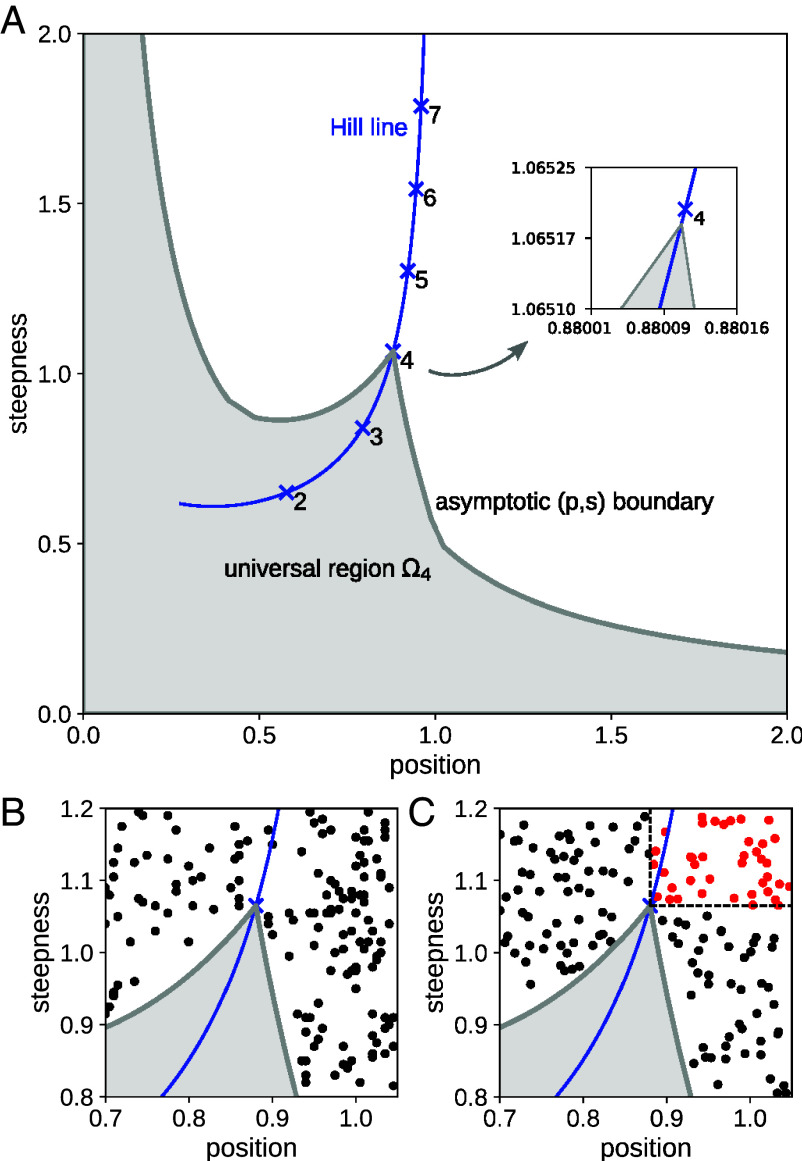
Universal position-steepness region and the Hopfield barrier. (*A*) Universal asymptotic (p,s) region, $\Omega_{4}$ (gray area), for thermodynamic equilibrium models with m=4 binding sites for the input ligand, with the Hill line shown as in Fig. 1*C*. The magnified view in the inset shows the cusp lying on the Hill line just below the (p,s) point for $H_{4}$. (*B*) Expanded view of the cusp showing (p,s) points lying outside the universal region (black dots) for rational functions which do not satisfy the coefficient condition, $\alpha_{i}\leq \beta_{i}$, for Eq. **9**. Only points outside $\Omega_{4}$ are shown for clarity. (*C*) Expanded view of the cusp showing (p,s) points lying outside the universal region for the graph with the hypercube structure $C_{4}$, output given by fractional saturation and parameter values chosen away from thermodynamic equilibrium. The red points beyond the dashed lines exceed $H_{4}$ in both position and steepness and confirm that the Hill function $H_{4}$ is the Hopfield barrier for the sharpness of models with m=4 input binding sites. Only points outside $\Omega_{4}$ are shown for clarity.

We elaborated the techniques previously introduced to estimate (p,s) regions like that in Fig. 1*C* (1, 10, 32) to plot the universal (p,s) region, $\Omega_{m}$, for input–output responses at t.e. with m input binding sites (Fig. 3*A*). The coefficients in Eq. **9** were sampled for l=m; the (p,s) points of the corresponding rational functions were plotted; and the resulting region was grown by biasing the sampling and expanding the parametric range so as to establish the asymptotic boundary. The algorithm is summarized in the *Materials and Methods* with further details in *SI Appendix*.

Fig. 3*A* shows $\Omega_{4}$ with confirmation of the asymptotic boundary shown in *SI Appendix*, Fig. S4. The asymptotic boundary of $\Omega_{6}$ is shown in *SI Appendix*, Fig. S5. These universal regions have the same characteristic properties satisfied by the $C_{4+1}$ model in Fig. 1*C*, as described previously. First, the regions have an asymptotic boundary. Second, the regions are effectively bounded in the positive quadrant. The wings that asymptote to the axes are more visible in Fig. 3*A*. Third, $\Omega_{m}$ has a cusp that falls on the Hill line and lies just below the (p,s) point of $H_{m}$ (Fig. 3*A*, *Inset*). During asymptotic convergence, the cusp approaches increasingly close to the (p,s) point of $H_{m}$ as the parametric range is increased (*SI Appendix*, Figs. S4 and S5). As before, the Hill function acts as a sharpness barrier at t.e.: while there are input–output responses with either higher position or higher steepness than $H_{m}$, there are none with both higher position and higher steepness.

The coefficient constraints for Eq. **9**, which characterize equilibrium input–output responses (*SI Appendix*), are required for these properties of $\Omega_{m}$. If rational functions are permitted which do not obey the constraints, then (p,s) points can be found that lie outside the universal region (Fig. 3*B*). This confirms that the asymptotic region arises only for equilibrium input–output responses. As a further check on the universality of $\Omega_{m}$, we calculated the (p,s) regions for six specific models with m=4 input binding sites and found them all to be contained within $\Omega_{4}$, as expected (*SI Appendix*, Fig. S6). Details of the models are given in *SI Appendix*.

The situation is profoundly different away from t.e., as mentioned previously. We considered the graph with hypercube structure $C_{4}$ and the input–output response given by fractional saturation, which is the average number of bound inputs normalized to the total number of input binding sites (here 4). When edge labels are allowed to be away from t.e., we readily found (p,s) points that lie outside $\Omega_{4}$ (Fig. 3*C*). In particular, we found (p,s) points that are greater in both position and steepness than those of $H_{4}$ (Fig. 3*C*, red points), thereby confirming that $H_{4}$ is the universal Hopfield barrier for sharpness of input–output responses with m=4 input binding sites. There is nothing special about the structure $C_{4}$: other graph structures with m=4, such as those in *SI Appendix*, Fig. S6, can yield (p,s) points lying outside $\Omega_{4}$ and exceeding $H_{4}$ in both position and steepness, when edge labels are allowed to be away from t.e.

The tapering wings of $\Omega_{m}$ (Fig. 3*A*) can be understood as follows. Recall the unnormalized versions of steepness and position, $s^{u}\left(r\right)$ and $p^{u}\left(r\right)$, which satisfy Eq. **12**. We can distinguish two extreme cases, when $p^{u}\left(r\right)\ll x_{0.5}$, so that $p\left(r\right)=p^{u}\left(r\right)/x_{0.5}$ is small, while $s\left(r\right)=s^{u}\left(r\right)x_{0.5}$ can become large, or when $x_{0.5}\ll p^{u}\left(r\right)$, so that p(r) is large, while s(r) can become small. Input–output responses that satisfy these conditions appear not to be biologically meaningful.

### Failure of Universality Away From Thermodynamic Equilibrium.

As discussed above, the universality of $\Omega_{m}$ breaks down completely away from t.e. This strikingly different behavior may be understood in terms of the difference between the vectors μ(G) at t.e. (defined through Eq. **3**) and ρ(G) away from t.e. (Eq. **2**), in terms of which s.s. probabilities are calculated by normalizing (Eq. **4**). For a given vertex i∈ν(G), $\mu_{i}\left(G\right)=\mu \left(P\right)$, where P is a path from 1 to i, with the cycle condition ensuring that this value is independent of the chosen path. It is this property of path independence that ultimately shows, through coarse-graining, that the degree of x in $\mu_{i}\left(G\right)$ is given simply by the number of input binding sites that are bound by the input in vertex i (*Materials and Methods*). This degree is essentially independent of the structure of the graph G. It then readily follows that the rational structure of input–output responses at t.e., as described in Eq. **9**, is also essentially independent of G.

Away from t.e., however, we must use ρ(G), rather than μ(G), to calculate s.s. probabilities. An input–output response is still a rational function of x but, as Eq. **2** makes clear, $\rho_{i}\left(G\right)$ depends on all spanning trees rooted at i. In consequence, the degree of x in $\rho_{i}\left(G\right)$ now depends crucially on the structure of G and becomes unrelated to the number of bound sites in i (*SI Appendix*). For hypercube graphs of structure $C_{m}$, input–output responses away from t.e. have degree $2^{m}-1$ in x (1) and can therefore have substantially higher sharpness than responses at t.e. (25). It is this marked difference in rational structure that underlies Fig. 3*C* and explains the failure of universality away from t.e.

Very little is known about the shape of (p,s) regions away from t.e., which, as just explained, are now model dependent. Numerical estimation of regions beyond quite small graphs is hampered in part by the combinatorial intractability of Eq. **2**. What little evidence there is ref. 1 suggests that nonequilibrium (p,s) regions also have a cusp on the Hill line, just below the (p,s) point for $H_{z}$ where z is an integer. However, it is an open problem to understand how z depends on the underlying graph.

## Discussion

We have provided here a rigorous biophysical justification for the Hill functions. As pointed out previously, they have been widely exploited in biology for over a century, for both data fitting and modeling. Yet, they have been nothing other than a convenient family of rational functions. We have shown by numerical calculations for m=4 (Fig. 3) and m=6 (*SI Appendix*) that Hill functions with integer Hill coefficients are the universal Hopfield barriers for sharpness of input–output responses: given any Markov process model with m input binding sites at t.e., no matter how complicated, the sharpness of any input–output response (Eq. **5**) lies within the universal, model-independent (p,s) region $\Omega_{m}$, and cannot be higher in both position and steepness than that of the Hill function $H_{m}$ (Fig. 3*A*). In contrast, if any such graph is away from t.e., then input–output responses can be found whose position and steepness both exceed those of $H_{m}$ (Fig. 3*C*). A. V. Hill could not have anticipated, at the time he introduced his eponymous functions (9), their deep connection to thermodynamics.

Our numerical results strongly suggest that the conclusions described above hold for all values of m, and it remains an open problem to give a mathematical proof of this. Considerable subtlety arises because of the shape of $\Omega_{m}$. It is not true in general that the position or steepness of an input–output response is less than the position or steepness, respectively, of $H_{m}$ but both assertions become true within the cusp (Fig. 3). Position and steepness become increasingly tightly constrained within the cusp so as to asymptotically fall on the Hill line itself. The precise nature of this changing constraint is not yet understood, and this appears to be one of the main barriers to a proof.

The winged, cuspidal shape of $\Omega_{m}$ (Fig. 3*A*) is particularly tantalizing. Its universality suggests that it may have some deeper mathematical significance that has yet to be understood. Perhaps this may encourage mathematicians to examine more closely a mathematical object that has emerged directly from biology. There remains much work to be done, as noted above, to understand the sharpness regions of input–output responses away from t.e. Another important question arises in moving beyond the reservoir assumptions made here. Biological ligands are always present in limited amounts and may be engaged in other activities beyond the system of interest. Such issues have been largely ignored in the literature but evidence is emerging as to the consequences of doing so (33, 34). Ligand limitation and distraction can be accommodated within the linear framework (28) but the resulting input–output responses begin to stray outside the elegant confines of rational functions.

Our results illustrate the significance of Hopfield’s insights into energy expenditure, as first put forward for biosynthetic error correction (2) and then elaborated, as explained previously, for any form of information processing (1). No matter what information processing task is being undertaken, there is a fundamental limit—the Hopfield barrier—to how well it can be carried out at t.e. The limit is set by fundamental physics, in effect by the cycle condition. Energy expenditure has been widely studied in areas like pattern formation, force generation, and active matter (35, 36), but its role in information processing has been more elusive. This may reflect the fact that, in areas other than information processing, the relevant Hopfield barriers are zero: for example, directed movement is impossible at t.e. Information processing, in contrast, can certainly occur at t.e., even though, as Hopfield recognized, evolution has bypassed the Hopfield barriers.

We believe the time is now ripe to analyze in more depth the functional impact of energy expenditure in cellular information processing. Previous studies have suggested putative Hopfield barriers (23, 37–39) and there is now growing evidence for the significance of nonequilibrium functionality in gene regulation (10, 28, 40, 41). Much insight could be gained by characterizing the Hopfield barriers for the various information processing tasks undertaken by cells, as we have done here for the sharpness of input–output responses. Such a research programme may not only bring to light some of the general principles at work in biology but may also reveal further objects of mathematical interest. Moreover, the method of coarse-graining used here, which is generally applicable, leads us to ask whether similar universality and model independence may also be found for other Hopfield barriers. We hope the results of the present paper will stimulate further studies of Hopfield barriers in cellular information processing.

## Materials and Methods

### Master Equation and Eq. **2**.

A linear framework graph, G, gives rise to a linear dynamics as follows: each edge may be thought of as a chemical reaction under mass-action kinetics with the edge label as the rate constant. Since an edge has only a single source vertex, the dynamics must be linear. It may be written in matrix form as

[13] $$\begin{array}{c} \frac{du(t)}{\mathrm{dt}}=L\left(G\right)\cdot u\left(t\right)\;, \end{array}$$

where u(t) is the vector of vertex probabilities at time t and L(G) is the Laplacian matrix of G (42). Under reservoir assumptions, Eq. **13** is the master equation of the corresponding Markov process (12, Theorem 4). A s.s. of Eq. **13** must lie in the kernel of L(G), which is one-dimensional when G is strongly connected. The canonical basis element ρ(G)∈ kerL(G) is calculated by using the Matrix-Tree theorem of graph theory, which relates the minors of L(G) to spanning trees of G. This gives Eq. **2**, from which the s.s. can be calculated by normalizing to remove the proportionality constant, as in Eq. **4**.

### Coarse-Graining.

This method was introduced in ref. 31. Let G be any strongly connected, reversible graph. Choose any partition of the vertices into disjoint subsets: $\nu \left(G\right)=G_{1}\cup \cdots \cup G_{s}$ and $G_{w}\cap G_{z}=\emptyset$ when w≠z. A *coarse-grained* graph, C(G), is constructed on the vertices 1,⋯,s, corresponding to the subsets of the partition. There is an edge $w\to_{C(G)}z$ if, and only if, there is an edge $i\to_{G}j$ for some vertex $i\in G_{w}$ and some vertex $j\in G_{z}$. C(G) thereby inherits reversibility from G. The edge labels on C(G) are given by

[14] $$\begin{array}{c} \ell \left(w\to_{C(G)}z\right)=Q\left(\sum_{j\in G_{z}}\rho_{j}\left(G\right)\right)\;, \end{array}$$

where ρ(G) is the vector defined in Eq. **2**. The quantity Q in Eq. **14** is chosen to ensure that the labels have dimensions of (time)^−1^, but its actual value is irrelevant because, with these labels, C(G) satisfies the cycle condition, even when G does not. Hence, as far as s.s. probabilities of C(G) are concerned, only the label ratios are relevant (Eq. **4**), so that Q cancels out. The key point is that, with the labeling in Eq. **14**, the coarse-graining formula in Eq. **6** holds. The choice of labels in Eq. **14** is essentially unique if C(G) has to satisfy the cycle condition and Eq. **6** has to hold. Note that this coarse-graining is only at s.s. and nothing is implied about the dynamics of C(G).

### Rational Structure and Eqs. **8** and **9**.

The independence of $\chi_{i}$ in Eq. **8** from x arises because, if $j\in G_{S}$, then $\mu_{j}\left(G\right)=\gamma_{j}x^{\left|S\right|}$, where |S| is the size of S, or the number of input binding sites that are bound by the input, and $\gamma_{j}$ is independent of x. To see this, recall that the reference vertex, 1, in G was chosen to be a state in which no input ligand is bound. Take any path, P, of reversible edges from 1 to j, $P:1=i_{1}⇋\cdots ⇋i_{k}=j$. It follows from the definition of μ(P) in Eq. **3** that traversing P from $i_{1}$ to $i_{k}$, forward edges may be encountered at which the input binds, which each contribute a factor x to μ(P), as well as forward edges at which the input unbinds, which each contribute a factor $x^{-1}$ to μ(P). Since no ligand is bound in $i_{1}=1$ and $j\in G_{S}$ has |S| input binding sites, the net effect of the bindings and unbindings along P must be to contribute exactly $x^{\left|S\right|}$ to μ(P). Provided G satisfies the cycle condition, μ(P) is independent of the choice of P. Hence, $\mu_{j}\left(G\right)=\mu \left(P\right)=\gamma_{j}x^{\left|S\right|}$, where $\gamma_{j}$ does not depend on x. It then follows that x occurs to the same degree in both the numerator and each term of the denominator of Eq. **8**, so that it cancels out and $\chi_{i}$ is independent of x, as claimed.

The denominator of $u_{i}^{*}$ in Eq. **4** is now a polynomial in x of degree l, where l is the maximum number of input binding sites that are bound by the input ligand. Also, every degree less than l must occur in the denominator, since states are formed by successive binding of the input ligand. Hence, from Eq. **5**, r(x) is a rational function whose denominator polynomial is of degree l in x, as shown in Eq. **9**, with $\beta_{i}>0$ for 0≤i≤l. Since the numerator of $u_{j}^{*}$ in Eq. **4** is always part of the denominator, it follows from Eq. **5** that $0\leq \alpha_{i}\leq \beta_{i}$.

### Determination of the Universal (*p*,s) Region in Fig. 3A.

Parameters are sampled as follows. Eq. **9** has 2(m+1) parameters (coefficients) for graphs at t.e. with m input binding sites. The denominator parameters, $\beta_{i}$, are sampled by choosing $\log_{10}\beta_{i}$ uniformly at random in the interval [−a,a], for a fixed exponent range a. Having chosen the $\beta_{i}$, the logarithm of the numerator parameters, $\log_{10}\alpha_{i}$, are sampled uniformly at random in the interval $\left[-a,\log_{10}\beta_{i}\right]$, to satisfy the constraints in Eq. **9**. As previously found (10), the boundary of the (p,s) region stabilizes rapidly as a is increased (*SI Appendix*, Fig. S4) to give an asymptotic boundary.

Boundaries are estimated as follows. The two-dimensional (p,s) space is divided into a grid of small square cells of side length 0.005. The current working boundary is defined by those cells which contain sampled (p,s) points but which have only empty cells above or below in the same column or to the left or right in the same row. The working boundary is then recomputed in two phases. First, each of the sampled parameter sets that yield (p,s) points on the working boundary is repeatedly “mutated” by randomly choosing new parameter values near the sampled value, independently for each parameter, until a parameter set is found whose (p,s) point goes into an empty cell. This may generate a new working boundary. Second, for each sampled (p,s) point on the resulting boundary, a target point is determined that lies outside the boundary and repeated mutations are attempted, as before, to reduce the distance in (p,s) space to this target point. This second phase is important to avoid becoming trapped in deep valleys during the first phase. The algorithm is considered to converge when no new boundary cells are created after a number of iterations that is specified as a hyperparameter; we took it to be 1,500.

## Supplementary Material

Appendix 01 (PDF)

## Data Availability

Source codes have been deposited in Zenodo (43–45).
