# Supplementary material for: The Hill function is the universal Hopfield barrier for sharpness of input–output responses
Source: Proc Natl Acad Sci U S A. 2024 May 24;121(22):e2318329121. doi: 10.1073/pnas.2318329121 (PMC11145184; doi:10.1073/pnas.2318329121)
Supplement: Supplementary file 1 — Appendix 01 (PDF) [file pnas.2318329121.sapp.pdf]

Supporting Information for

The Hill function is the universal Hopfield barrier for sharpness of input-output responses

Rosa Martinez-Corral, Kee-Myoung Nam, Angela DePace and Jeremy Gunawardena

Corresponding Author Jeremy Gunawardena.
E-mail: jeremy_gunawardena@hms.harvard.edu

This PDF file includes:

Supporting text
Figs. S1 to S6
Tables S1 to S2
SI References

Supporting Information Text

We follow the notation and terminology of the main text, using s.s. for steady state and t.e. for thermodynamic equilibrium.

Input-output responses. Table S1 shows example input-output responses from a variety of biological contexts. Similar *dose responses* are also commonly measured in toxicology, where the inputs are diverse chemicals and outputs can be at the organism level (1).

Type	System	Input	Output	Refs.
enzymes	ATCase	aspartate	activity	(2)
	PAN	ATP	activity	(3)
GPCRs	mGluR	glutamate	current	(4)
	aortic tissue	5-HT	contraction	(5)
ion channels	KCNQ2	activator	current	(6)
	AChR	photoactivatable agonist	current	(7)
transporters	haemoglobin	oxygen	frac. sat.	(8)
	CBG	dexamethasone	frac. sat.	(9)
	rubredoxin	protons (pH)	avg. site binding	(10)
signalling	MLCK	Ca^{2+}-calmodulin	activity	(11)
	PKA	cAMP	frac. sat. & activity	(12)
	Ste5	α-factor	luminescence	(13)
gene regulation	λ *cI*	CI	lacZ reporter	(14)
	hunchback	Bicoid	Hb protein	(15)

Table S1. Experimentally measured input-output responses. For the meaning of an acronym for a system, see the corresponding citation. Avg.: average; frac. sat.: fractional saturation.

Examples of linear framework graphs. Linear framework graphs can represent different forms of molecular complexity (16), as illustrated in Fig.S1. The product construction in Fig.S1A shows how the hypercube structure, $\mathcal{C}_m$, can be defined as

$$\mathcal{C}_m = \underbrace{\mathcal{C}_1 \times \cdots \times \mathcal{C}_1}_{m \text{ times}} .$$

The product construction can be applied to any two graph structures; see (16) for details. Hierarchical nesting, which arose in studying allostery (18), is another procedure, akin to the product construction, that can generate increasingly complex graph structures.

Eq. 9 gives all input-output responses at t.e. Suppose given a rational function $r(x)$ of degree l, defined as in Eq. 9 of the main text with the accompanying restrictions on the coefficients α_i and β_i. We show here that labels can be assigned to the graph structure $\mathcal{C}_l$ so that it can reach t.e., with x being the concentration of the unique binding ligand, and coefficients λ_i can be found, as in Eq.5 of the main text, such that $r(x)$ is the resulting input-output response.

There is a standard labelling for hypercube structures at t.e. that was introduced in (19, SI, §3), which should be consulted for more details. Set theory notation is needed to explain this labelling. Each vertex of the hypercube structure corresponds to a subset of bound sites. Let $S \subseteq \{1, \cdots, l\}$ denote that subset and use this symbol as the index for that vertex. The edges of $\mathcal{C}_l$ are then given by $S \leftrightharpoons S \cup \{i\}$ where $i \notin S$. At t.e., only the label ratios are needed to determine s.s. probabilities. Let the association constant, $K_{i,S}$, denote the ratio,

$$K_{i,S} = \frac{1}{x}\left(\frac{\ell(S \to S \cup \{i\})}{\ell(S \cup \{i\} \to S)}\right) ,$$

so that $xK_{i,S}$ is the label ratio. The $K_{i,S}$ have dimensions of $(\text{concentration})^{-1}$ and there are $l2^{l-1}$ such association constants. Whatever unit is used for concentration, we will assume it is the same one used in Eq.9 in the main text. Because of the cycle condition, the association constants are not independent. Restricting to those $K_{i,S}$ for which i is less than all the sites in S, denoted $i < S$, yields an independent and complete set of $2^l - 1$ parameters for $\mathcal{C}_l$ at t.e. Note that, since the empty set, $\emptyset$, has no members, the condition $i < \emptyset$ is trivially satisfied for all sites i and so the association constant, $K_{i,\emptyset}$, for binding to site i with no other sites bound, is one of the independent parameters. For convenience, we will refer to the resulting graph as $\mathcal{C}_l$.

In terms of this parameterisation, the vector $\mu(\mathcal{C}_l)$ is given as follows. Suppose that $S = \{i_1, \cdots, i_k\}$ is a vertex of $\mathcal{C}_l$ for which the sites are written in order, so that $i_1 < i_2 < \cdots < i_k$. Then, the vertex S may be reached from the empty subset, $\emptyset$, by a path of reversible edges such that,

$$\mu_S(\mathcal{C}_l) = K_{i_k,\emptyset} K_{i_{k-1},\{i_k\}} \cdots K_{i_1,\{i_2,\cdots,i_k\}} x^k .$$

By construction, these association constants are included in the independent set of parameters. It is now straightforward to determine the s.s. probability of vertex S from Eq.4 of the main text. It follows that the denominator in Eq.4 of the main text is

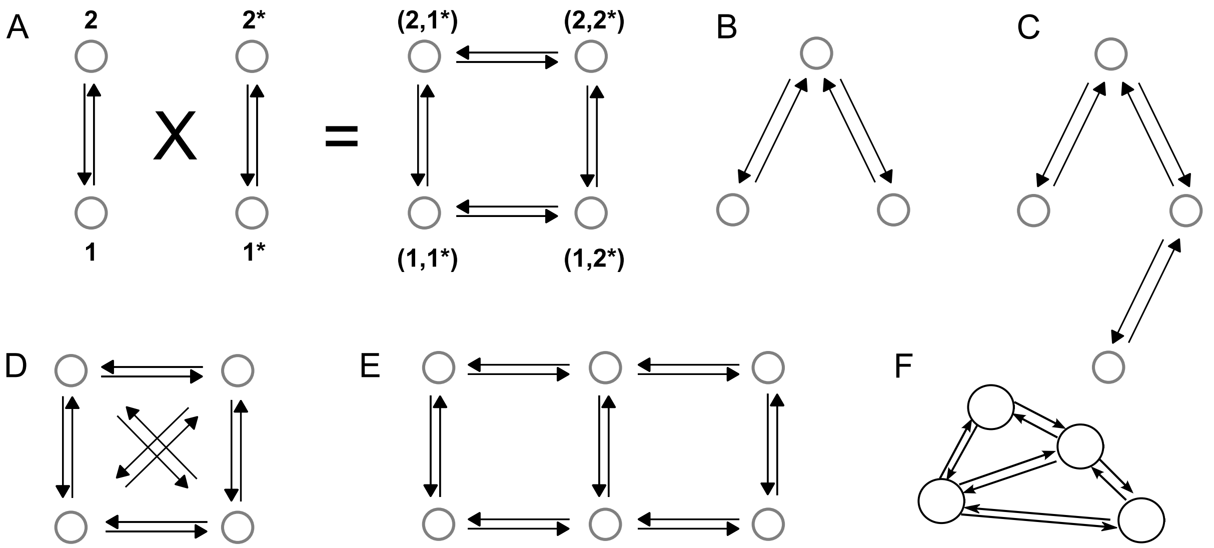

Fig. S1. Graph structures; labels are omitted for clarity. **A.** Product construction, with the vertices indexed to illustrate how the hypercube structure, $\mathcal{C}_2$, arises as the product of 2 copies of $\mathcal{C}_1$. **B.** Graph structure that could represent mutual exclusion, such as two binding sites which are sufficiently close together that only one site can be bound at a time. **C.** Graph structure that could represent ordering, such as two binding sites in which binding first to site 1 allows binding to site 2 but binding first to site 2 excludes binding to site 1. **D.** Graph structure that could represent 4 options which can arbitrarily interchange, such as methylation patterns on a lysine residue. **E.** Lattice graph structure in which the vertices could represent conformations or discrete spatial locations. **F.** Graph structure representing the conformational ensemble of the pentameric, ligand-gated ion channel GLIC, constructed by Markov State Modelling, adapted from (17).

given by

$$\sum_{0\leq k\leq l}\left(\sum_{1\leq i_1<\cdots<i_k\leq l} K_{i_k,\emptyset}K_{i_{k-1},\{i_k\}}\cdots K_{i_1,\{i_2,\cdots,i_k\}}\right)x^k\,. \quad [1]$$

The term in Eq.1 with $k=0$, for which there is an empty product between the brackets, is taken by convention to be 1. Returning to the rational function $r(x)$ in Eq.9 of the main text, we may assume, by dividing above and below by β_0, that $\beta_0=1$. There are l remaining coefficients in the denominator, $\beta_1,\cdots,\beta_l$, which are given. Since there are 2^l-1 association constants, which exceeds l as soon as $l>1$, it is straightforward to recursively choose them to yield the correct denominator polynomial. We will illustrate this for $l=3$, from which the reader should be able to readily see the general procedure. We need to give values to the $7=2^3-1$ independent association constants, $K_{1,\emptyset}$, $K_{2,\emptyset}$, $K_{3,\emptyset}$, $K_{1,\{2\}}$, $K_{1,\{3\}}$, $K_{2,\{3\}}$ and $K_{1,\{2,3\}}$, and choose the $8=2^3$ λ_S coefficients for the input-output response. Comparing Eq.1 to the denominator of Eq.9 in the main text, we first require that

$$K_{1,\emptyset}+K_{2,\emptyset}+K_{3,\emptyset}=\beta_1\,. \quad [2]$$

Choose $K_{1,\emptyset}$ and $K_{2,\emptyset}$ arbitrarily so that $0<K_{1,\emptyset}+K_{2,\emptyset}<\beta_1$, which we may always do, and then choose $K_{3,\emptyset}$ to satisfy Eq.2. Next we require that

$$K_{1,\{2\}}K_{2,\emptyset}+K_{1,\{3\}}K_{3,\emptyset}+K_{2,\{3\}}K_{3,\emptyset}=\beta_2\,. \quad [3]$$

As before, we may choose $K_{1,\{2\}}$ and $K_{1,\{3\}}$ arbitrarily so that,

$$0<K_{1,\{2\}}K_{2,\emptyset}+K_{1,\{3\}}K_{3,\emptyset}<\beta_2\,,$$

which we may again always do. The key point here, which makes the recursion work, is that the quantities to be chosen satisfy a linear equation (Eq.3) in which the previously chosen quantities give the coefficients. We may now choose $K_{2,\{3\}}$ to satisfy Eq.3. Finally, we require that

$$K_{1,\{2,3\}}K_{2,\{3\}}K_{3,\emptyset}=\beta_3\,,$$

which we may easily satisfy by choosing $K_{1,\{2,3\}}=\beta_3/(K_{2,\{3\}}K_{3,\emptyset})$. This determines the 7 association constants. Now choose $\lambda_\emptyset=\alpha_0$, $\lambda_{\{1\}}=\lambda_{\{2\}}=\lambda_{\{3\}}=\alpha_1/\beta_1$, $\lambda_{\{1,2\}}=\lambda_{\{1,3\}}=\lambda_{\{2,3\}}=\alpha_2/\beta_2$ and $\lambda_{\{1,2,3\}}=\alpha_3/\beta_3$. Because of the restriction that $0\leq\alpha_i\leq\beta_i$, as stipulated for Eq.9, it follows that $0\leq\lambda_S\leq1$, as required for Eq.5. It is not difficult to see that we recover $r(x)$ from these choices and that a similar procedure works in general for any $l>1$. This completes the proof.

Intrinsic measures of sharpness. The rational functions defined by Eq.9 can be substantially more complicated than sigmoidal input-output responses. They may have positive basal values at $x = 0$ and be non-monotonic with multiple peaks and troughs and they may asymptote as $x \to \infty$ to a value other than their maximum (Fig.S2). Non-monotonic responses occur in areas like pharmacology and toxicology; particularly intricate examples are found in pH titrations of biomolecules (10). Fig.S2 shows an example based on Eq.9 of the main text, which also illustrates how the normalisation value, $x_{0.5}$, in Eq.10 of the main text is chosen.

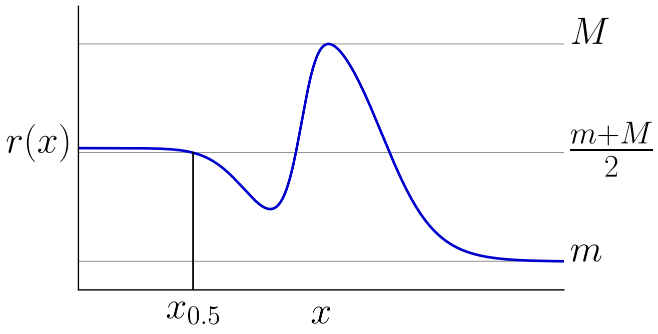

Fig. S2. Rational function and normalisation. The plot shows an example input-output response, $r(x)$, obtained from Eq.9 for $l = 6$, with the following coefficients, $\alpha_0 = 5, \alpha_1 = 50, \alpha_2 = 0.1, \alpha_3 = 10, \alpha_4 = 0.01, \alpha_5 = 1, \alpha_6 = 0.001, \beta_0 = 10, \beta_1 = 100, \beta_2 = 100, \beta_3 = 10, \beta_4 = 0.01, \beta_5 = 1, \beta_6 = 0.01$.

We asserted in the main text that the supremum of $|dq/dy|$ is attained for a finite value of y. To see this, note from the structure of the rational function $r(x)$ in Eq.9 of the main text that $dq/dy = dr/dx \cdot x_{0.5}$ must asymptote to the y-axis at infinity, $\lim_{y\to\infty} dq/dy = 0$. Hence, if the supremum of $|dq/dy|$ is only found asymptotically when $y \to \infty$, it must be the case that $dq/dy = 0$ for all $y \in [0, \infty)$. Hence, $q(y)$ is a constant and therefore so too is $r(x)$. But this is impossible because the input ligand must bind to at least one site, so that the degree l in Eq.9 satisfies $l \geq 1$ and $r(x)$ cannot be a constant.

Estimating position and steepness. Numerical estimation of position and steepness was carried out differently depending on whether or not the underlying model is at t.e. For an equilibrium model, the coefficients of the rational function in Eq.9 of the main text can be algebraically calculated. Away from equilibrium, this is no longer feasible because of the combinatorial explosion described in the main text. Instead, s.s. probabilities were estimated directly from the Laplacian matrix in Eq.13 of the main text by singular value decomposition. The code for (p, s) calculations is available in the GitHub repository github.com/rosamc/universal-boundaries-Hopfield-barrier.git. The ranges and densities of points used here were determined by trial-and-error, to the point where further increases did not significantly change (p, s) values.

Equilibrium models. To calculate (p, s) values for equilibrium models quickly and accurately, we implemented a custom algorithm in C++, called from Python using `pybind11`, using high-precision floating-point types provided by the GNU MPFR library through the Boost interface (www.boost.org). After extensive testing, we found similar results for 50 or 100 digit precision, so 50 digit precision was used. The algorithm works as follows.

Depending on how a particular model is parameterised, which is discussed further below, the coefficients of the rational function $r(x)$ in Eq.9 are calculated in terms of these parameters. Unless $(m(r) + M(r))/2 = 0.5$, which is the case for the fractional saturation input-output response for Model I (below), $r(x)$ is evaluated at the points $x = 10^{-60+k.0.02}$ for $k = 0, 1, \cdots, 5999$. The quantities $m(r)$ and $M(r)$ in Eq.10 are estimated and $x_{0.5}$ is determined by finding the smallest positive solution to the polynomial equation, $r(x) = (m(r) + M(r))/2$. Polynomial solving was performed with a custom C++ implementation of the Aberth-Ehrlich root-finding method (20, 21). The code returns complex roots. Roots having a positive real part, and an imaginary part with absolute value smaller than 10^{-15} are considered to be real zeros, while the other roots are discarded. This polynomial solving code is available at github.com/kmnam/polynomials.git.

Having calculated $x_{0.5}$, the normalised function $q(y) = r(yx_{0.5})$ is obtained by multiplying α_i and β_i in Eq.9 by $(x_{0.5})^i$. The derivatives dq/dy, d^2q/dy^2 and d^3q/dy^3 are then calculated algebraically. The roots of d^2q/dy^2 are estimated by polynomial solving, using the algorithm just described. The local maxima and minima of dq/dy are determined by evaluating the sign of d^3q/dy^3 at each root and the maximum of $|dq/dy|$ for $y \in [0, \infty)$ is determined. Eq.11 of the main text then gives the (p, s) values.

Non-equilibrium model in Fig. 3C. Because of the combinatorial explosion away from t.e., as discussed in the main text, it is not computationally feasible to determine the coefficients of the rational function $r(x)$ in Eq. 9 in terms of the model parameters. We therefore numerically evaluated the input-output response and estimated (p, s) values using finite differences.

To calculate $r(x)$ at a given x we used singular value decomposition (SVD) directly on the matrix $\mathcal{L}(G)$, described in Eq.13 of the main text. SVD gives a basis for $\ker \mathcal{L}(G)$, from which the s.s. probabilities can be calculated, as in Eq.4 of the main text, and $r(x)$ thereby determined. This was again done in C++ using 100-digit precision floating-point types provided by the MPFR library through the Boost interface, with the BDCSVD routine from the Eigen library. This code is available in github.com/rosamc/universal-boundaries-Hopfield-barrier.git.

For accuracy, it is necessary to evaluate $r(x)$ over a large range and at many values of x, which makes the calculation slow. To balance computation time against accuracy, we followed a two-step approach. First, we get an initial estimate of $x_{0.5}$. The rational function $r(x)$ is sparsely evaluated at the points $x = 10^{-60+k\times1.62}$ for $k = 0, \cdots, 74$. Then we use the `interp1d` function in the Python Scipy library to interpolate values of $r(x)$ at $x = 10^{-60+k\times0.0012}$ for $k = 0, \cdots, 99999$. We use these interpolated values to estimate $m(r)$, $M(r)$ and $x_{0.5}$ using Eq. 10 in the main text.

From this initial estimate of $x_{0.5}$, we re-evaluate $r(x)$ over a smaller range more densely. We calculate $r(x)$ at 10^3 logarithmically-spaced points between $10^{-4} \times x_{0.5}$ and $10^3 \times x_{0.5}$ and interpolate values of $r(x)$ at 10^5 logarithmically-spaced points within this range, $i_1, i_2, \cdots, i_{10^5}$. We then calculate a more accurate $x_{0.5}$. The value of $|dr/dx|$ is calculated by finite differences as $|(r(i_{k+1}) - r(i_k))/(i_{k+1} - i_k)|$ for $k = 1, \cdots, 10^5 - 1$ and the maximum value and corresponding input point, i_m, are obtained. This gives the un-normalised steepness $s^u(r)$, and the un-normalised position, $p^u(r)$, as defined in the main text, from which the (p, s) values are calculated by $p(r) = p^u(r)/x_{0.5}$ and $s(r) = s^u(r)x_{0.5}$.

Numerically estimating (p, s) regions. We explain here in more detail the procedure used to estimate the universal (p, s) regions Ω_4 (Fig.3A and Fig.S4) and Ω_6 (Fig.S5) and the (p, s) regions for various specific models (Fig.1C, Fig.S6). The basic idea is to use iterative, biased sampling of the relevant parameters, whose values are drawn from a parametric range $[10^{-a}, 10^a]$, to reach a boundary for that value of a. Boundaries are calculated for increasing values of a and an asymptotic boundary is considered to have been reached when the boundaries for two consecutive a values coincide when plotted together; see Fig.S4 for Ω_4 and Fig.S5 for Ω_6. Experience has shown that the algorithm can get trapped prematurely and the procedures outlined below have been refined by trial-and-error specifically to overcome this problem. The method was originally developed in (19) and subsequently elaborated in (22). The Python code for the boundary procedure is available in the GitHub repository github.com/rosamc/GeneRegulatoryFunctions.git.

Generalities. (p, s) regions are estimated within some rectangular box within the positive quadrant, which determines the extent of (p, s) space to be explored. Unless otherwise noted, position was explored from 0.4 to 2.5, and steepness from 0.3 to 2.5. The box is divided into a grid of square "cells" of width and height 0.005 in position and steepness. (p, s) regions are extended in an iterative manner from an initial seed region. A grid cell is considered to be filled when a (p, s) point falls within it; a filled cell is on the boundary of the current region if one of its four immediate neighbours (above, below, left or right) is not filled.

The parametric range for the relevant parameters is chosen as $[10^{-a}, 10^a]$ for some value of $a > 0$. The sequence of a values used to establish an asymptotic boundary depends on the (p, s) region being estimated and is given in Table S2. The relevant parameters may involve constraints, which are always in the form of inequalities. For example, for the universal region Ω_m, the relevant parameters are the coefficients, $\alpha_0, \cdots, \alpha_m$ and $\beta_0, \cdots, \beta_m$, of the rational function in Eq.9, and these are subject to the constraint that $\alpha_i \leq \beta_i$. Other parametric constraints arise for some of the models described below. We will refer to those parameters, like β_i, that lie within a semi-infinite interval, as "independent", and the others, like α_i, whose range is limited to a finite interval by the independent parameters, as "dependent". The search starts by randomly sampling parameter sets from the specified range, ensuring the constraints are satisfied, until 10 grid cells are filled (see Initialisation below). Only one parameter set is kept for each filled cell. The boundary is subsequently extended by modifying the parameter sets within boundary cells and iteratively exploring the parameter space to grow the region until the final boundary is reached. The algorithm described below does not necessarily give rise to a boundary that is always a simple closed curve, with a clearly defined inside and outside, but we encountered no difficulties in this respect in practice.

During the boundary estimation, only the grid cell corresponding to a (p, s) point is recorded, alongside the corresponding parameter set, not the exact (p, s) value. Once the final boundary is reached, Mathematica is used to recalculate the (p, s) values for those parameter sets on the boundary, thereby providing an independent test of the final boundary. The Mathematica algorithm paralleled the one described previously: definition of $r(x)$ from the parameter values, evaluation over $[10^{-60}, 10^{60}]$, calculation of $x_{0.5}$, definition of $q(y)$, calculation of derivatives, calculation of the extrema of dq/dy. The Mathematica function `Solve` was used to determine the roots of polynomials and the function `D` was used to calculate derivatives. The Mathematica code is available in github.com/rosamc/universal-boundaries-Hopfield-barrier.git.

A (p, s) point calculated by Mathematica is retained to calculate the definitive boundary only when it falls in the original grid cell for the corresponding parameter values. Otherwise, the point is discarded. In the vast majority of cases there was excellent agreement, with most of the discrepancies arising when the Mathematica-calculated point was in a grid cell next to the original one (see Further details below).

The search process is delimited by hyperparameters. Some of them are fixed throughout: *niters_conv* = 1500; *niters_conv_points* = 1000; *niters_target* = 500; *L_project* = 15; *tol_target* = 0.001. Some of them are varied between runs: *prob_par* = 0.2, 0.5; *prob_replace* = 0.2, 0.6; *extremesu* = $[-2, 2]$, $[-1.5, 1.5]$, $[-1, 1]$, $[-0.5, 0.5]$. (The meaning of these hyperparameters is given in the description of the search algorithm below.) We run the boundary estimation for each of the $16 = 2 \times 2 \times 4$ combinations of the three varying hyperparameters, starting in each case from a different seed region (below) to avoid trapping. There were differences in the rate of convergence, with a few, rare cases failing to converge in the wall time allowed, or experiencing cluster-related impediments (Table S2). For those that converged, we never noticed any substantial difference among the boundaries computed with different hyperparameter combinations, suggesting the boundary has been accurately estimated in each case. In order to estimate the final boundary, we took the parameter sets corresponding to boundary cells for each of the converged runs and calculated their (p, s) value using Mathematica as explained above. The points where the Mathematica-calculated (p, s) point fell in the original grid cell were collected together and the definitive boundary determined from this collection obtained from all converged runs (in which some cells may no longer be on the boundary). To draw the definitive boundary shown in the figures, the `alphashape` routine of the Python Alpha Shape toolbox (https://pypi.org/project/alphashape) was used,

with a boundary-specific, manually-adjusted alpha parameter to ensure a good match to the input (p, s) points. The C++ code, Mathematica code and the Python Jupyter notebooks used to determine the boundaries and make the figure plots are available in github.com/rosamc/universal-boundaries-Hopfield-barrier.git.

Initialisation. Having defined the grid and specified the search hyperparameters, as outlined above, the first step is to identify a few parameter sets whose (p, s) values fall within different grid cells. For this, parameter values are randomly chosen from the specified parametric range, subject to the appropriate inequality constraints. For example, for the universal models, the $\log_{10}\beta_i$ are chosen uniformly at random within the range $[-a, a]$, and the $\log_{10}\alpha_i$ are then chosen uniformly at random in the range $[-a, \log_{10}\beta_i]$, so as to satisfy the constraint $\alpha_i \leq \beta_i$. Constraints for other models are treated similarly. A (p, s) value is calculated as described previously and the corresponding grid cell is considered filled. If a (p, s) value falls into an already filled cell, the new parameter set is discarded and the old one retained. The process is repeated until 10 different cells are filled and the corresponding current working boundary is defined as above.

Extension. After initialisation is completed, the current working boundary is iteratively extended until it stabilises. The extension procedure involves slightly changing the parameter sets of the cells on the current working boundary, with the intention that these new parameter sets will yield slightly different (p, s) values, hopefully corresponding to empty cells outside the current working boundary. At each iteration, the parameter sets are changed in different ways in order to explore their neighbourhood, through the *Mutation* and *Pulling* steps explained below. The boundary is assumed to have stabilised when no new boundary cell is filled for *niters_conv* consecutive iterations. If a cell remains at the boundary for *niters_conv_points* consecutive iterations, then it is considered to be on the final boundary and the *Mutation* and *Pulling* steps are omitted for that cell. This reduces the computational cost of the procedure in case the boundary converges fast in a given region but not another.
Mutation. At each iteration, new parameter sets are generated and tested for their ability to produce a (p, s) point that fills a cell that is either outside or at the current boundary. If the filled cell is at the current boundary, the old parameter set is replaced by the new one with probability *prob_replace*. This stochastic replacement of boundary points helps to escape local trapping: by slightly changing parameter sets on the boundary, it may become possible to move outside the boundary in subsequent iterations. For each boundary parameter set, the algorithm attempts at most 20 trials to modify its value and generate a new accepted parameter set, whose cell is either outside or at the current boundary. Trials are halted as soon as a new parameter set is accepted. After applying the mutation procedure to all boundary parameter sets, the current working boundary is recomputed.
To generate a new parameter set from an old one, each parameter value v is replaced by z with probability *prob_par*, where z is obtained from v in a different way depending on the trial number, as specified below. The hyperparameter *prob_par* stochastically controls by how much a given parameter set is changed: the larger value of 0.5 is suitable for quick exploration, while the smaller value of 0.2 helps to fine tune the boundary near stabilisation. Let the value of the hyperparameter *extremesu* be [fmin, fmax] (with fmin < 0, fmax > 0), and let $\mathcal{N}(\mu, \sigma)$ be the normal distribution with mean μ and standard deviation σ.

- trials 1-2: choose θ randomly from the uniform distribution on [fmin, fmax] and set $z = v \times 10^{\theta}$.
- trials 3-5: choose z randomly from the normal distribution $\mathcal{N}(v, v/0.1)$;
- trials 6-9: choose z randomly from the normal distribution $\mathcal{N}(v, v/0.5)$;
- trials 10-13: choose z randomly from the normal distribution $\mathcal{N}(v, v)$;
- trials 14-15: choose z randomly from the normal distribution $\mathcal{N}(v, v/2)$;
- trials 16-20: choose θ randomly from the uniform distribution on [fmin/0.5, fmax/0.5] and set $z = v \times 10^{\theta}$.

Note that this procedure can result in parameter values outside the allowed parametric range $[10^{-a}, 10^{a}]$ or the range determined by the constraints, as described above. If a parameter choice z lies outside the desired parameter range $[c, d]$, then the nearest value to z on the boundary of the range is chosen instead: if $z < c < d$, then c is chosen, while if $c < d < z$, then d is chosen. This can be important to find parameter combinations that lead to extreme regions of the (p, s) space. If there are constraints among the parameters, then the independent parameters, as defined above, are chosen first and the dependent parameters chosen afterwards. If a dependent parameter is not selected for change because of the stochasticity associated with *prob_par* but one of the independent parameters that determines its constraint is selected, the dependent parameter may no longer satisfy its constraint. If so, this dependent parameter is changed by the smallest amount that allows its constraint to be satisfied.
Pulling. After applying the *Mutation* step, a different strategy is used of "pulling" towards a target grid cell, t, that is chosen for each boundary cell c, so as to lie outside the current boundary. Each cell is assigned integer coordinates based on its position in the grid. If the grid has K rows and L columns, the coordinates are the corresponding row and column numbers chosen in $[0, K-1] \times [0, L-1]$. Two kinds of targets are defined, one to pull away from the boundary and one to pull away from the centre of the region.
For the first target, the nearest boundary cell, b, is identified that is no more than 3 cells to the right of c. This target is only used if there are more than 100 cells at the boundary in order to have a good chance of finding b. If no such cell exists, c is skipped. If b is found, the line joining the integer coordinates of c and b is rotated around the coordinates of c by an angle of $\pi/4$ away from the current boundary. The cell that lies farthest on this line, while remaining within the grid and no farther than *L_project* cells away, is chosen as t.

Model	Range	# Runs	# Correct	# (0-2)%	# [2-5)%	Other
Ω_4 (Fig.3)	0.3	16	9	7	0	-
	0.5	16	0	16	0	-
	0.7	16	0	16	0	-
	1	16	0	16	0	-
	2	16	0	15	1	-
	3	16	0	15	1	-
lower right	3	16	0	0	0	†
upper left	3	16	9	7	0	-
lower left	3	16	0	16	0	‡
Ω_6 (Fig.S5)	0.3	16	0	16	0	-
	0.5	16	0	3	13	-
	0.7	12	0	1	11	-
	1	11	0	1	10	-
	2	16	0	6	10	-
	3	16	0	2	14	-
I (Fig.S6)	1	16	16	0	0	-
	2	16	16	0	0	-
	3	16	16	0	0	-
	5	15	15	0	0	-
	7	16	16	0	0	-
II (Fig.S6)	0.5	14	14	0	0	-
	1	15	15	0	0	-
	2	16	16	0	0	-
	3	16	16	0	0	-
	6	16	16	0	0	-
III (Fig.S6)	1	16	0	4	12	-
	2	16	0	9	7	-
	3	16	0	11	5	-
	4	16	0	12	4	-
IV (Fig.S6)	3	16	16	0	0	-
	5	16	16	0	0	-
	7	16	16	0	0	-
	9	16	16	0	0	-
	10	0	N/A	N/A	N/A	-
V (Fig.S6)	0.5	16	16	0	0	-
	1	16	16	0	0	-
	2	16	16	0	0	-
	3	16	15	1	0	-
	6	16	12	4	0	-
VI (Fig.S6)	0.5	16	16	0	0	-
	1	15	15	0	0	-
	2	16	16	0	0	-
	3	15	15	0	0	-
	6	16	16	0	0	-

Table S2. Details of the boundary estimation runs. The columns record the type of model (Model); the parametric range exponent, a, in $[10^{-a}, 10^{a}]$ (Range); the number of successfully completed runs (# Runs); the number of those completed runs in which all boundary points calculated by Mathematica were in the original cell (# Correct); the number of completed runs with less than 2% of boundary points in a neighbouring cell to the original ($(0-2)$%); the number of completed runs with at least 2% and no more than 5% of boundary points in the neighbouring cell ($[2-5)$%); some other possibility that is explained in the Notes to this caption (Other). For the various parts of Ω_4, see Fig.S3. The $a = 10$ analysis for Model IV was exceptionally slow and failed to converge. As there was little difference between the regions for $a = 7$ and $a = 9$, $a = 10$ was abandoned. Notes. (†) — there were 13 runs with $[0.2 - 2.2)$% of points for which Mathematica was unable to find the roots of the polynomials; 3 runs with $[0.2 - 0.5)$% of non-neighbouring cell points; 13 runs with $[9 - 15)$% neighbouring cells; and 3 runs with $[15 - 18)$% neighbouring cells. (‡) — there were 12 runs with $[0.1 - 1)$% of points for which Mathematica was unable to find the roots of the polynomials; and 5 runs with $[0.1 - 0.2)$% of non-neighbouring cell points.

For the second target, the centroid, ζ, of the filled cells is calculated. The line joining the integer coordinates of ζ and c is extended away from the boundary. The cell that lies farthest on this line from c, while being outside the boundary and remaining within the grid and no farther than Euclidean distance *L_project*, is chosen as t.
Having chosen a target cell t for a given boundary cell c, the exploration proceeds as follows. A new parameter set is generated from the parameter set for c, as described above for trial 1 of the mutation step. If the Euclidean distance from the (p, s) of this new parameter set to the bottom left corner (p, s) of t is reduced, the old parameter set is replaced with the new one. If not, the old parameter set is retained and used again. The process is iterated until either the new distance to t is less than *tol_target* or the number of iterations reaches *niters_target*. If the resulting parameter set fills a new cell, the boundary will have been extended. After visiting all cells on the current boundary, a new working boundary is computed.

Further details. Boundary estimation for the universal Ω_4 region in Fig. 3 was done in parts to reduce the memory requirements (Fig. S3). We first identified the parametric range corresponding to the asymptotic boundary for the central region, we then extended the "wings", and we finally explored the lower-left region of the quadrant (Table S2). Let $[c, d, f, g]$ denote the rectangular box between position values $[c, d]$, where $c < d$, and steepness values $[f, g]$, where $f < g$. The boundary lying in the central box $[0.4, 2.5, 0.3, 2.5]$ was estimated with cells of size 0.005, as described above. The boundaries for the parametric ranges $[10^{-2}, 10^{2}]$ and $[10^{-3}, 10^{3}]$ overlapped, so we took the latter range as the one corresponding to the asymptotic boundary. Then, we estimated the "wings" lying in the boxes $[1.2, 2.1, 0, 0.4]$ (lower right) and $[0, 0.5, 0.8, 2.1]$ (upper left), using the same range of $[10^{-3}, 10^{3}]$. For each of these two wing boxes, we seeded the search with the boundary points identified in one of the runs for the central box, which fell within the respective wings. Finally, to double check this extension and ensure that we could extend the boundary down to small (p, s) values, we also estimated the boundary in the box $[0, 1.5, 0, 1.8]$ (lower left), using a coarser grid of cells of size 0.01, again to reduce the memory cost of the calculation.

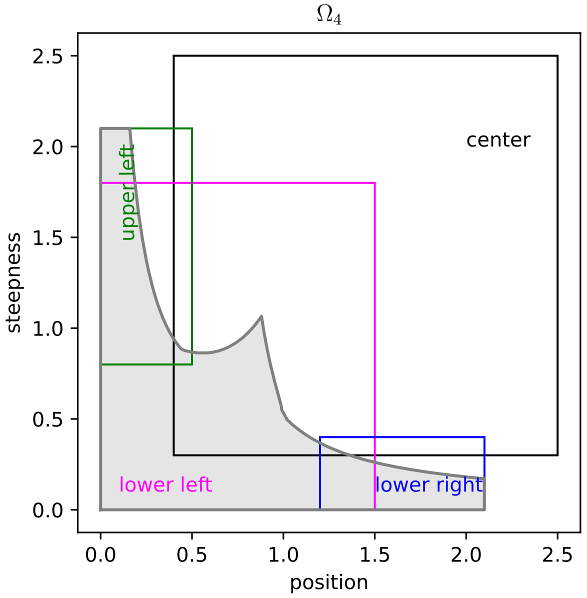

Fig. S3. Grid boxes used to estimate the universal region Ω_4, as described in the text.

Table S2 gives details of the boundary estimation for the various models considered in the paper. As noted above, cluster-related issues sometimes led to unfinished runs or the wall time allocated was insufficient. Since the majority of the jobs finished, we did not repeat the searches for the few that did not. Similarly, in some cases, the (p, s) values calculated by Mathematica for boundary parameter sets did not fall in the same cell (Table S2). These parameter sets were discarded and not used to calculate the final boundary. It can be seen from Table S2 that when Mathematica values are non-coincident, the corresponding (p, s) values nearly always fall in a neighbouring cell, with the exceptions being only for the lower-right and lower-left boxes of Ω_4. For the universal models, Ω_4 and Ω_6, the non-coincident points are always far from the cusp on the Hill line, typically in regions of high position and low steepness. For Model III, in contrast, the non-coincident points are on the boundary of the region, at high values of steepness, but these points are always far from the boundary of Ω_4.

Six specific models. As a partial test of the universal (p, s) region Ω_4, we estimated the asymptotic region for six previously-studied models at t.e., each with 4 input binding sites, and confirmed that they all fell within Ω_4 (Fig.S6). In the descriptions which follow, we use G to denote the corresponding graph.

Model I. This model is the *average binding* gene-regulation model analysed in (19). Considered more generally, the graph structure is the hypercube $\mathcal{C}_n$ for $n = 4$, representing the binding of a single ligand to n sites, and the output is *fractional saturation*, or the

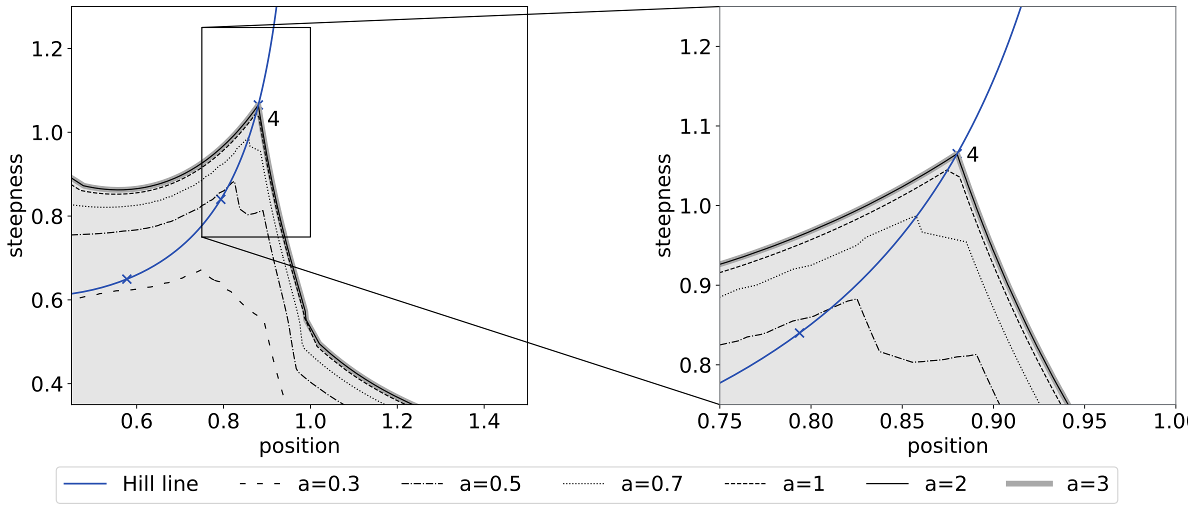

Fig. S4. Universal region Ω_4, showing convergence to the asymptotic boundary for increasing parametric ranges, $[10^{-a}, 10^{a}]$, shown as varying linestyles, as indicated in the key. The same conventions are followed as in Fig.3A of the main text, with the (p, s) value for $\mathcal{H}_4$ marked. The right-hand panel shows in greater resolution the box around the cusp in the left-hand panel.

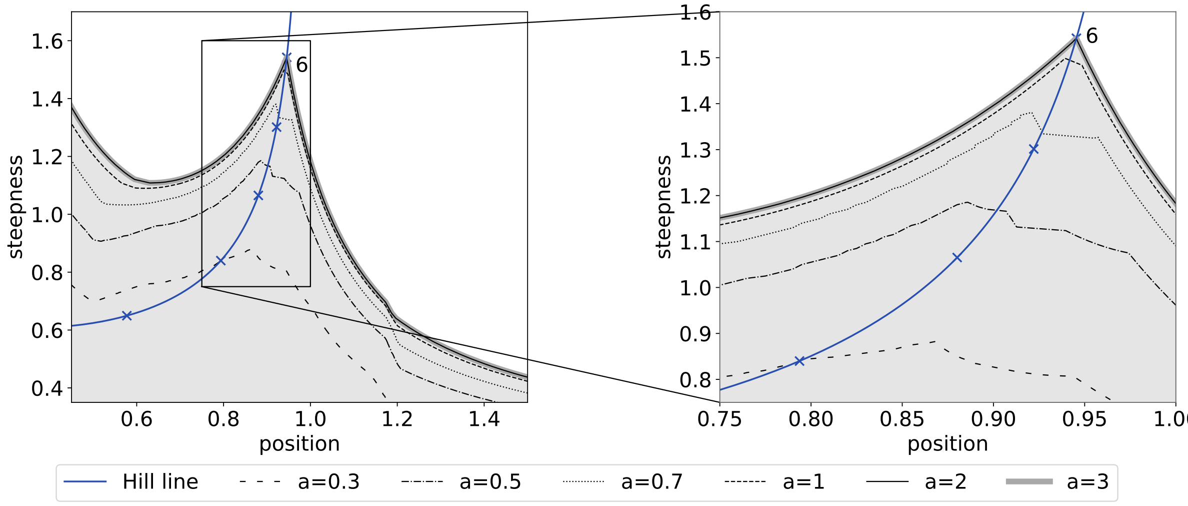

Fig. S5. Universal region Ω_6, showing convergence to the asymptotic boundary, following the same format as in Fig.S4.

average fraction of bound sites. Using the set theory notation described above, let $|S|$ denote the number of elements in the subset S. The average binding input-output response is given by,

$$r(x) = \frac{1}{n}\left(\sum_{S \subseteq \{1,\cdots,n\}} |S| u_S^*(G)\right) .$$

In (19), the ligand is a transcription factor (TF) binding to DNA and the output represents the normalised level of mRNA expression. The parameters are the independent association constants, $K_{i,S}$ with $i < S$, introduced previously.
The Model I region forms a substantially reduced part of Ω_4, although it still exhibits the cusp that approaches the (p, s) value of $\mathcal{H}_4$ (Fig.S6).

Model II. This model is the extended gene-regulation model analysed in (15). The graph structure is the hypercube $\mathcal{C}_{n+1}$ for $n = 4$, representing the binding of one ligand, A, to n binding sites and the binding of a second ligand, B, to a single additional site (Fig.1A). In (15), A is a TF and B is RNA Polymerase being recruited to its promoter site.
It will be helpful for Models V and VI below to consider the more general situation of two ligands, A and B, with the input A binding to a sites $1, \cdots, a$ and the co-regulator B binding to b sites $a+1, \cdots, a+b$. The graph structure is then the product $\mathcal{C}_{a+b} = \mathcal{C}_a \times \mathcal{C}_b$ (16). The vertices can be denoted by the ordered pair, (S, T), where $S \subseteq \{1, \cdots, a\}$ and $T \subseteq \{a+1, \cdots, a+b\}$.

Independent association constants can be found for this model by following the spanning tree procedure described in (19),

$$K_{i,(S,\emptyset)} \text{ for } i \in \{1,\cdots,a\} \setminus S,\ i < S$$
$$K_{i,(S,T)} \text{ for } i \in \{a+1,\cdots,a+b\} \setminus T,\ i < T\,.$$

The first set of association constants are for A binding only in the presence of A, while the second set of association constants are for B binding in the presence of both A and B. All other association constants can be rationally expressed in terms of these independent ones (19).

For Model II, $a = n = 4$ and $b = 1$ and the output is the probability of B being bound when at least one site is bound by A. The input-output response for the resulting graph G is therefore given by,

$$r(x) = \sum_{\emptyset \neq S \subseteq \{1,\cdots,n\}} u^*_{(S,\{n+1\})}(G)\,. \qquad [4]$$

To fix the concentration of B, we chose $K_{n+1,(\emptyset,\emptyset)}[B] = 1$.

Gene-regulation functions are sometimes found to increase monotonically with increasing TF concentration (15) and we were curious about the impact of such monotonicity on the (p,s) region. We therefore sought parametric conditions that would ensure this behaviour. We show below that if the affinity of B binding increases with the number of A sites that are bound, irrespective of which sites are bound, so that $K_{n+1,(S,\emptyset)} > K_{n+1,(T,\emptyset)}$ whenever $|S| > |T|$, then $r(x)$ increases monotonically with x. We imposed this constraint, which leads to the asymptotic (p,s) region shown for Model II in Fig.S6. Interestingly, the Model II region matches Ω_4 better than does Model I, except when position is high and steepness is low.

Model III. This model is a variation of Model II in which the monotonicity constraints on the parameters were dropped and the output was altered to allow for a response when no A is bound, so that,

$$r(x) = \sum_{\emptyset \subseteq S \subseteq \{1,\cdots,n\}} u^*_{(S,\{n+1\})}(G)\,.$$

Fig. S6 shows that the asymptotic region is now quite different from that for Model II. The region has a poorly-defined cusp that is not on the Hill line and is far from the (p,s) value of $\mathcal{H}_4$. This appears to be due to the choice of $[B]$ being set by taking $K_{n+1,(\emptyset,\emptyset)}[B] = 1$. If $K_{n+1,(\emptyset,\emptyset)}[B]$ is taken to be sufficiently large, the region acquires a well-defined cusp on the Hill line that approaches $\mathcal{H}_4$, as in Fig.1C in the main text, which shows the (p,s) region for this model with $K_{n+1,(\emptyset,\emptyset)}[B] = 1000$. Model III reveals the impact on the (p,s) region of apparently small changes in the input-output response and also the influence of other ligands, a topic that deserves further study.

Model IV. This model is the Monod-Wyman-Changeaux model for allostery (23), as analysed using coarse graining in (24). A biomolecule is assumed to exist in two inter-converting conformations, to which a ligand binds at $m = 4$ sites. We follow the original parameterisation, with L being the label ratio for inter-conversion of the conformations with no ligand bound, and K_1 and K_2 being the association constants for binding to each conformation independent of the site or the pattern of binding at other sites. The output is fractional saturation, as in Model I. The input-output response is given by the Monod-Wyman-Changeux formula (24, Eq.55),

$$r(x) = \frac{xK_1(1+xK_1)^{m-1} + xK_2L(1+xK_2)^{m-1}}{(1+xK_1)^m + L(1+xK_2)^m}\,.$$

The asymptotic (p,s) region shows a clear cusp on the Hill line which approaches $\mathcal{H}_4$ (Fig.S6). The upper boundary of the region runs close to the Hill line and the lower part of the region is substantially restricted.

Model V. This model considers the binding of an activator A to sites $1,\cdots,a$, and the binding of a repressor B to sites $a+1,\cdots,a+b$, using the notation introduced for Model II above. Here, $a = 4$ and $b = 2$. Each vertex (S,T) is taken to influence the output by the amount $\iota(S,T) = \max(0, |S| - |T|)$ and the input-output response is taken to be the fractional average of this quantity,

$$r(x) = \frac{1}{a}\left(\sum_{(S,T)} \iota(S,T)u^*_{(S,T)}(G)\right)\,.$$

For this model, we took $[B] = 1$ in the same concentration units used for the association constants. The asymptotic (p,s) region for this model matches Ω_4 less well than Model II and fails to extend to regions of higher position.

Model VI. This model is a variant of Model V in which the repressor B is considered to be dominant over A and all A sites have to be occupied for output to occur. The graph is the same and the concentration of B was fixed in the same way but the input-output response is now taken to be,

$$r(x) = u^*_{(\{1,\cdots,a\},\emptyset)}(G)\,.$$

It is interesting that the asymptotic (p,s) region for this model is quite similar to that for Model V, despite the substantial difference in the output.

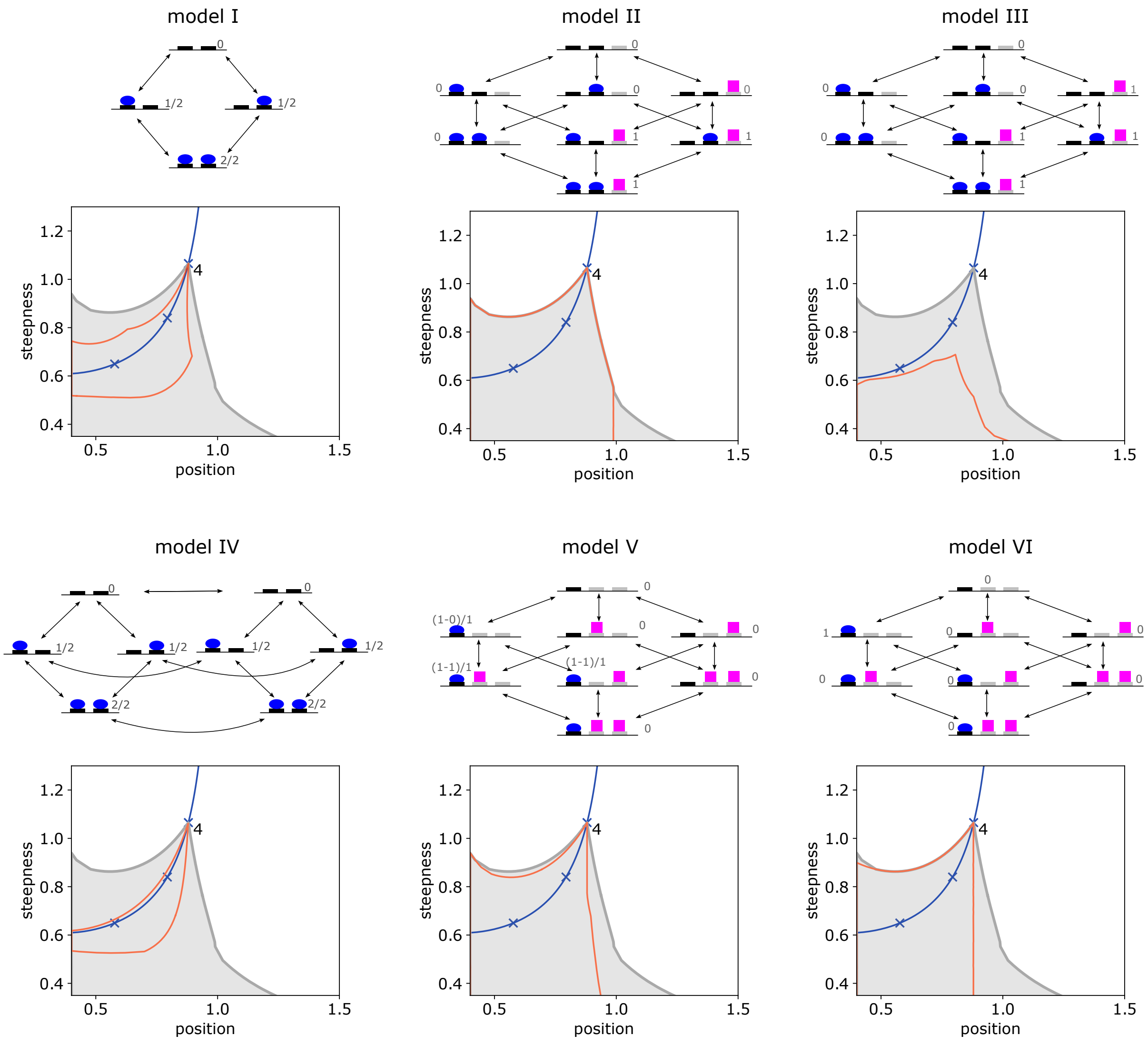

Fig. S6. Asymptotic (p, s) regions for 6 specific models with $m = 4$ input binding sites. The red curve is the asymptotic boundary of each model, with the gray region showing the universal region Ω_4. The same conventions are used as for Fig.3A in the main text, with the (p, s) value of $\mathcal{H}_4$ marked at the cusp of Ω_4. In each case the model region is contained within Ω_4, as expected for universality. The graphs above each plot describe the corresponding model with the number of input binding sites taken to be 2 (Models I-IV) or 1 (Models V and VI), rather than 4, for ease of visualisation. The reversible edges are shown as bidirectional arrows and vertices are marked by expressions that give the corresponding weight in the input-output response.

Monotonicity of the input-output response for Model II. In Model II above, we restricted the input-output response, $r(x)$, to be a monotonically strictly increasing function of x, so that $r(x) < r(y)$ whenever $x < y$. We prove here the parametric conditions that result in this behaviour. We require two simple observations.

Lemma 1. Suppose $a_0, \cdots, a_n$ and $b_0, \cdots, b_n$ are positive numbers. If a_k/b_k does not decrease with k, so that $a_k/b_k \leq a_l/b_l$ when $k < l$, then the rational function

$$r(x) = \frac{a_0 + a_1 x + \cdots + a_n x^n}{b_0 + b_1 x + \cdots + b_n x^n}$$

does not decrease for $x > 0$. If a_k/b_k also increases at some k, so that $a_k/b_k < a_{k+1}/b_{k+1}$, then $r(x)$ increases strictly for $x > 0$.

Proof. The numerator of the derivative of $r(x)$ has the form

$$u(x) = \left(\sum_{1 \leq i \leq n} i a_i x^{i-1}\right)\left(\sum_{0 \leq j \leq n} b_j x^j\right) - \left(\sum_{1 \leq j \leq n} j b_j x^{j-1}\right)\left(\sum_{0 \leq i \leq n} a_i x^i\right)$$

and the sign of $u(x)$ determines the monotonicity of $r(x)$. If $u(x) \geq 0$ for $x > 0$, then $r(x)$ is non-decreasing for $x > 0$ and if $u(x) > 0$ for $x > 0$, then $r(x)$ is strictly increasing for $x > 0$. We may reorganise the expression for $u(x)$ so that,

$$u(x) = \left(\sum_{1 \leq i \leq n} i b_0 a_i x^{i-1}\right) - \left(\sum_{1 \leq j \leq n} j a_0 b_j x^{j-1}\right) + \left(\sum_{1 \leq i,j \leq n} i a_i b_j x^{i+j-1}\right) - \left(\sum_{1 \leq i,j \leq n} j b_j a_i x^{i+j-1}\right)$$

Collecting together the same powers of x, the first two terms yield the expression

$$\sum_{1\leq i\leq n} i(b_0a_i - a_0b_i)x^{i-1}\,. \qquad [5]$$

Doing the same for the last two terms, we see that the resulting expression can be broken into two parts,

$$\left(\sum_{1\leq j<i\leq n} (ia_ib_j - jb_ja_i)x^{i+j-1}\right) + \left(\sum_{1\leq i<j\leq n} (ia_ib_j - jb_ja_i)x^{i+j-1}\right)\,,$$

where the terms corresponding to $i = j$ have cancelled. Since the indices i and j are arbitrary, we are at liberty to exchange them in the sum on the right and collect the two sums together to yield,

$$\sum_{1\leq j<i\leq n} (ia_ib_j - jb_ja_i + ja_jb_i - ib_ia_j)x^{i+j-1} = \sum_{1\leq j<i\leq n} (i-j)(a_ib_j - b_ia_j)x^{i+j-1}\,. \qquad [6]$$

Because the a's and b's are all positive, it follows from Eqs.5 and 6 that, if $x > 0$ and a_k/b_k does not decrease with k, then $u(x) \geq 0$ and that if a_k/b_k increases for some k, then $u(x) > 0$ for all $x > 0$. The result follows, as claimed.

There is a minor extension of Lemma 1 which will be helpful below. If the numerator polynomial in $r(x)$ has no constant term, so that $a_0 = 0$, but all the other a's and b's remain positive, then the expression in Eq.5 becomes positive. Moreover, at no stage in the remainder of the proof is it necessary to divide by a_0. Hence, even if the weaker conditions of the Lemma hold, and a_k/b_k does not decrease with k, the fact that $a_0 = 0$ ensures that $r(x)$ increases strictly for $x > 0$.

Lemma 2. Suppose I and J are arbitrary finite index sets and that a_i, b_i and A_j, B_j are arbitrary positive quantities indexed over I and J, respectively. If $a_i/b_i \leq A_j/B_j$ for any $i \in I$ and any $j \in J$, then

$$\frac{\sum_{i\in I} a_i}{\sum_{i\in I} b_i} \leq \frac{\sum_{j\in J} A_j}{\sum_{j\in J} B_j}\,.$$

If the former inequality is strict for some i and j, then the latter inequality is also strict.

Proof. The inequality in the statement of Lemma 2 is equivalent to

$$\left(\sum_{j\in J} A_j\right)\left(\sum_{i\in I} b_i\right) - \left(\sum_{i\in I} a_i\right)\left(\sum_{j\in J} B_j\right) \geq 0\,.$$

Rearranging the expression on the left gives,

$$\sum_{i\in I, j\in J} (A_jb_i - a_iB_j)\,.$$

Since $a_i, b_i, A_j, B_j > 0$, this expression is non-negative if $A_j/B_j \geq a_i/b_i$ for all $i \in I$ and $j \in J$. Furthermore, if $A_j/B_j > a_i/b_i$ for some i, j, the expression is positive. The result follows.

We can now return to the input-output response for Model II, as given in Eq.4. Recall from Eq.4 in the main text that s.s. probabilities are determined by the vector $\mu(G)$ at t.e. We can express the components $\mu_{(S,T)}(G)$ in terms of the independent parameters introduced above for Model II as follows. Note that, for the case we are considering, in the notation used above, $a = n$ and $b = 1$. Given any subset, $S \subseteq \{1, \cdots, n\}$, with $|S| = k$, suppose that the indices of S are written in increasing order, $S = \{i_1, \cdots, i_k\}$ with $1 \leq i_1 < i_2 < \cdots < i_k \leq n$, and let K_S denote the product of independent association constants,

$$K_S = K_{i_1,\{i_2,\cdots,i_n\}}K_{i_2,\{i_3,\cdots,i_n\}}\cdots K_{i_n,\emptyset}\,.$$

It then follows that,

$$\begin{aligned} \mu_{(S,\emptyset)}(G) &= K_Sx^{|S|} \\ \mu_{(S,\{n+1\})}(G) &= K_{n+1,(S,\emptyset)}K_Sx^{|S|}[B]\,, \end{aligned} \qquad [7]$$

where $x = [A]$. This covers all the vertices of G. If we assemble the following two polynomials in x,

$$f(x) = \sum_{\emptyset\neq S\subseteq\{1,\cdots,n\}} \mu_{(S,\{n+1\})}(G) \quad \text{and} \quad g(x) = \sum_{\emptyset\subseteq S\subseteq\{1,\cdots,n\}} \mu_{(S,\emptyset)}(G) + K_{n+1,(\emptyset,\emptyset)}[B]\,,$$

then Eq.4 of the main text shows that the input-output response in Eq.4 takes the form,

$$r(x) = \frac{f(x)}{g(x) + f(x)}\,.$$

It is easy to see that $r(x)$ is increasing if, and only if, $f(x)/g(x)$ is increasing. This rational function falls under the scope of Lemma 1 and the minor extension discussed after the proof, in which the constant term in the numerator is 0. Accordingly, we must consider the ratio of the coefficients having the same power of x,

$$\frac{\sum_{|S|=k} \mu_{(S,\{n+1\})}(G)}{\sum_{|S|=k} \mu_{(S,\emptyset)}(G)},$$

and show that these increase with k for $1 \leq k \leq n$. This question now falls under the scope of Lemma 2. Choose $1 \leq k < l \leq n$ and apply Lemma 2 with the index set I being those subsets S with $|S| = k$ and the index set J being those subsets T with $|T| = l$. Then, it follows from Eq.7 that,

$$\frac{\mu_{(S,\{n+1\})}(G)}{\mu_{(S,\emptyset)}(G)} = K_{n+1,(S,\emptyset)}[B] \quad \text{and} \quad \frac{\mu_{(T,\{n+1\})}(G)}{\mu_{(T,\emptyset)}(G)} = K_{n+1,(T,\emptyset)}[B]\,.$$

By hypothesis, $K_{n+1,(S,\emptyset)} < K_{n+1,(T,\emptyset)}$ when $|S| = k < l = |T|$, so, applying Lemma 2, we can conclude that $r(x)$ is monotonically strictly increasing, as claimed. This completes the proof of monotonicity for the input-output response of Model II.

References

1. E Agathokleous, I Iavicoli, D Barceló, EJ Calabrese, Micro/nanoplastics effects on organisms: a review focusing on 'dose'. *J. Hazard. Mater.* **417**, 126084 (2021).
2. P England, C Leconte, P Tauc, G Hervé, Apparent cooperativity for carbamoylphosphate in Escherichia coli aspartate transcarbamoylase only reflects cooperativity for aspartate. *Eur. J. Biochem.* **222**, 775–780 (1994).
3. YC Kim, A Snoberger, J Schupp, DM Smith, ATP binding to neighbouring subunits and intersubunit allosteric coupling underlie proteasomal ATPase function. *Nat. Commun.* **6**, 8520 (2015).
4. J Levitz, et al., Mechanism of assembly and cooperativity of homomeric and heteromeric metabotropic glutamate receptors. *Neuron* **92**, 143–159 (2016).
5. JW Black, P Leff, NP Shankley, J Wood, An operational model of pharmacological agonism: the effect of E/[A] curve shape on agonist dissociation constant estimation. *Br. J. Pharmacol.* **84**, 561–571 (1985).
6. F Bai, et al., A statistical thermodynamic model for ligands interacting with ion channels: Theoretical model and experimental validation of the KCNQ2 channel. *Front. Pharmacol.* **9**, 150 (2018).
7. LD Chabala, AM Gurney, HA Lester, Dose-response of acetylcholine receptor channels opened by a flash-activated agonist in voltage-clamped rat myoballs. *J. Physiol.* **371**, 407–433 (1986).
8. T Clerbaux, P Gustin, B Detry, ML Cao, A Frans, Comparative study of the oxyhaemoglobin dissociation curve of four mammals: man, dog, horse and cattle. *Comp. Biochem. Physiol.* **106A**, 687–694 (1993).
9. T Murakami, Positive cooperativity of [3H]dexamethasone binding to chick corticosteroid-binding globulin. *Comp. Biochem. Physiol.* **100A**, 361–364 (1991).
10. A Onufriev, DA Case, GM Ullmann, A novel view of pH titration in biomolecules. *Biochemistry* **40**, 3413–9 (2001).
11. R Geguchadzea, et al., Quantitative measurements of Ca^{2+}/calmodulin binding and activation of myosin light chain kinase in cells. *FEBS Lett.* **557**, 121–4 (2004).
12. J Bubis, LD Saraswat, SS Taylor, Tyrosine-371 contributes to the positive cooperativity between the two cAMP binding sites in the regulatory subunit of cAMP-dependent protein kinase I. *Biochemistry* **27**, 1570–1576 (1988).
13. M Malleshaiah, V Shahrezaei, PS Swain, SW Michnick, The scaffold protein Ste5 directly controls a switch-like mating decision in yeast. *Nature* **465**, 101–5 (2010).
14. IB Dodd, et al., Cooperativity in long-range gene regulation by the λ CI repressor. *Genes Dev.* **18**, 344–54 (2004).
15. J Park, et al., Dissecting the sharp response of a canonical developmental enhancer reveals multiple sources of cooperativity. *eLife* **8**, e41266 (2019).
16. T Ahsendorf, F Wong, R Eils, J Gunawardena, A framework for modelling gene regulation which accommodates non-equilibrium mechanisms. *BMC Biol.* **12**, 102 (2014).
17. C Bergh, SA Heusser, R Howard, E Lindahl, Markov state models of proton- and pore-dependent activation in a pentameric ligand-gated ion channel. *eLife* **10**, e68369 (2021).
18. CH Robert, H Decker, B Richey, SJ Gill, J Wyman, Nesting: Hierarchies of allosteric interactions. *Proc. Natl. Acad. Sci. USA* **84**, 1891–5 (1987).
19. J Estrada, F Wong, A DePace, J Gunawardena, Information integration and energy expenditure in gene regulation. *Cell* **166**, 234–44 (2016).
20. O Aberth, Iteration methods for finding all zeros of a polynomial simultaneously. *Math. Comput.* **27**, 339–44 (1973).
21. LW Ehrlich, A modified Newton method for polynomials. *Commun. ACM* **10**, 107–8 (1967).
22. R Martinez-Corral, et al., Transcriptional kinetic synergy: a complex landscape revealed by integrating modelling and synthetic biology. *Cell Syst.* **14**, 324–39 (2023).
23. J Monod, J Wyman, JP Changeux, On the nature of allosteric transitions: a plausible model. *J. Mol. Biol.* **12**, 88–118 (1965).
24. JW Biddle, R Martinez-Corral, F Wong, J Gunawardena, Allosteric conformational ensembles have unlimited capacity for integrating information. *eLife* **10**, e65498 (2021).
